# *RUNX1* mutations contribute to the progression of MDS due to disruption of antitumor cellular defense: a study on patients with lower-risk MDS

**DOI:** 10.1038/s41375-022-01584-3

**Published:** 2022-05-03

**Authors:** Monika Kaisrlikova, Jitka Vesela, David Kundrat, Hana Votavova, Michaela Dostalova Merkerova, Zdenek Krejcik, Vladimir Divoky, Marek Jedlicka, Jan Fric, Jiri Klema, Dana Mikulenkova, Marketa Stastna Markova, Marie Lauermannova, Jolana Mertova, Jacqueline Soukupova Maaloufova, Anna Jonasova, Jaroslav Cermak, Monika Belickova

**Affiliations:** 1 Institute of Hematology and Blood Transfusion, Prague, Czech Republic; 2 First Faculty of Medicine, Charles University, Prague, Czech Republic; 3 Department of Biology, Faculty of Medicine and Dentistry, Palacky University, Olomouc, Czech Republic; 4 Faculty of Science, Charles University, Prague, Czech Republic; 5 International Clinical Research Center, St. Anne’s University Hospital, Brno, Czech Republic; 6 Czech Technical University, Prague, Czech Republic; 7 First Department of Medicine, First Faculty of Medicine, Charles University and General University Hospital, Prague, Czech Republic

**Keywords:** Risk factors, Myelodysplastic syndrome, Cancer genomics, Translational research

## Abstract

Patients with lower-risk myelodysplastic syndromes (LR-MDS) have a generally favorable prognosis; however, a small proportion of cases progress rapidly. This study aimed to define molecular biomarkers predictive of LR-MDS progression and to uncover cellular pathways contributing to malignant transformation. The mutational landscape was analyzed in 214 LR-MDS patients, and at least one mutation was detected in 137 patients (64%). Mutated *RUNX1* was identified as the main molecular predictor of rapid progression by statistics and machine learning. To study the effect of mutated *RUNX1* on pathway regulation, the expression profiles of CD34 + cells from LR-MDS patients with *RUNX1* mutations were compared to those from patients without *RUNX1* mutations. The data suggest that *RUNX1*-unmutated LR-MDS cells are protected by DNA damage response (DDR) mechanisms and cellular senescence as an antitumor cellular barrier, while *RUNX1* mutations may be one of the triggers of malignant transformation. Dysregulated DDR and cellular senescence were also observed at the functional level by detecting γH2AX expression and β-galactosidase activity. Notably, the expression profiles of *RUNX1*-mutated LR-MDS resembled those of higher-risk MDS at diagnosis. This study demonstrates that incorporating molecular data improves LR-MDS risk stratification and that mutated *RUNX1* is associated with a suppressed defense against LR-MDS progression.

## Introduction

Myelodysplastic syndromes (MDS) are a heterogeneous group of diseases with clonal hematopoiesis [1]. MDS patients are usually stratified into four risk groups according to their risk of transformation to acute myeloid leukemia (AML) by the International Prognostic Scoring System (IPSS) [2] or 5 risk groups by the Revised International Prognostic Scoring System (IPSS-R) [3]. Low- and intermediate-1 (INT-1) risk groups of IPSS and very low-risk, low-risk, and part of the intermediate-risk groups of IPSS-R are considered lower-risk MDS (LR-MDS) [4, 5]. Despite the more favorable prognosis, some LR-MDS patients progress rapidly [6].

Early identification of LR-MDS patients at risk of rapid progression is crucial for the initiation of effective treatment. In this context, numerous studies have mapped the genomic landscape in MDS patients to improve risk stratification and prognosis estimation. There has been a long-lasting effort to upgrade scoring systems by incorporating molecular features to give rise to IPSS-molecular [7–14]. However, no unified results have been generally accepted yet. The sole mutated gene included in the MDS classification by the World Health Organization is *SF3B1*, which is related to the percentage of ring sideroblasts in erythroid elements of bone marrow (BM) [15].

*RUNX1* is a frequently mutated gene in hematological malignancies and is associated with an adverse course of disease. This gene encodes a transcription factor that is critical for embryonic hematopoiesis and the development of megakaryocytes and platelets in adult hematopoiesis [16]. Mutations in this gene are related to thrombocytopenia. Somatic mutations were identified in MDS, AML, chronic myelomonocytic leukemia, acute lymphoblastic leukemia, and chronic myeloid leukemia [17, 18].

This study aimed to identify molecular markers at diagnosis that indicate the risk of rapid disease progression in LR-MDS patients. Transcriptome analysis was used to uncover signaling pathways involved in malignant transformation. We identified mutated *RUNX1* as the main molecular marker of rapid progression and described its effect on the disruption of the antitumor cellular response.

## Materials and methods

### Patient cohort

The study cohort consisted of 214 patients with de novo LR-MDS according to the IPSS. Forty-one patients (19%) progressed within 5 years. Progression was defined according to the revised International Working Group criteria [19]. All patients whose samples were used in this study provided signed informed consent forms. The study was approved by the Institutional Scientific Board and the IHBT Ethics Committee (EK 4/AZV CR/06/2017) and was performed in accordance with the ethical standards of the Declaration of Helsinki. The median age of the cohort was 65 years (range, 20.8–86.5 years). The median follow-up period was 33.4 months (range, 0.2–183.0 months), and 133 (62%) patients were still alive. Twenty-eight patients underwent hematopoietic stem cell transplantation (HSCT), and for the purposes of this study, they were followed until the date of HSCT. The patient characteristics are summarized in SI 1.

### Sequencing

Samples of BM or peripheral blood from diagnosis and, if available, from progression (90% of patients who progressed) were processed. Specific protocols for DNA and RNA isolation and detailed descriptions of targeted gene sequencing, Sanger sequencing, and RNA sequencing are reported in the Supplementary Methods.

#### Targeted gene sequencing

The sequencing library was prepared by the TruSight Myeloid Sequencing Panel Kit (Illumina, San Diego, CA, USA), which targets certain regions of 54 genes involved in hematological malignancies. NextGene software (SoftGenetics, State College, PA, USA) and an in-house pipeline were used for analysis of the output data. Variants were selected for further analysis if they met the following criteria: minimal coverage of 500x, Phred score greater than 35, and variant allele frequency (VAF) of ≥0.05. Variants were analyzed using 1000 Genomes, dbSNP, Varsome, ExAc, and other databases.

#### Sanger sequencing

Sanger sequencing was used to determine whether the mutations in *RUNX1* present at both diagnosis and progression with a VAF close to 0.5 were somatic or germline. We designed primer pairs for the amplification of exons 5–7, where these mutations were found by next-generation sequencing (NGS). Primer sequences are described in SI 2.

#### RNA sequencing

Seventy samples were sequenced (detailed in SI 3). For library preparation, the NEBNext Ultra II Directional RNA Library Prep Kit for Illumina (New England Biolabs, Ipswich, MA, USA) was used. The processed data were analyzed by DAVID 6.8 and String 11.0 online tools using functional enrichment and analysis of protein–protein interaction networks. Furthermore, the data were analyzed using Gene Set Enrichment Analysis (GSEA) in GSEA software 3.0.

### Machine learning

Two different techniques for the feature selection method applicable to Cox hazard models were used: stepwise backward feature selection and elastic network. Two different datasets were used: data1—binary mutational data and data2—the number of distinct mutations per gene. The details of the methods are given in the Supplementary Methods.

### Immunohistochemistry

BM formalin-fixed paraffin-embedded (FFPE) sections (from four LR-MDS patients without *RUNX1* mutation and three LR-MDS patients with *RUNX1* mutation) were stained with rabbit anti-human γH2AX primary antibody (phosphoSer139, polyclonal; Cell Signaling, Danvers, MA, USA) as described in [20].

### β-galactosidase detection

Six LR-MDS and six HR-MDS cryopreserved BM samples were thawed and washed in PBS with anti-clumping agent according to the manufacturer’s instructions (Gibco, Waltham, MA, United States). The cells were washed twice in autoMACS rinsing buffer (Miltenyi Biotec, Bergisch Gladbach, Germany) and incubated for 1 hour (37 °C, 5% CO_2_) with the β-galactosidase stain FITC (Senescence assay kit; Abcam, Cambridge, UK). Then, the cells were washed in PBS and stained for 30 min in a cocktail of antibodies specified in the Supplementary Methods. The cells were washed and directly measured at Cytek Aurora (Cytek, Fremont, CA, USA). The data were analyzed with the FlowJo software (BD, Franklin Lakes, NJ, USA).

### Statistical analysis

MedCalc (MedCalc Software Ltd, Ostend, Belgium) was used to perform a Kaplan–Meier survival analysis, Cox proportional hazard regression (for univariate and multivariate analyses), the Mann–Whitney test, Fisher’s exact test, and the chi-squared test. Graphs were created in GraphPad Prism 7 (GraphPad Software, La Jolla, CA, USA). Statistical level of significance was set at 0.05. Data were assumed to be non-normal (tested by Shapiro-Wilk test).

## Results

### Mutational landscape of LR-MDS patients and survival analyses

**Fig. 1:**
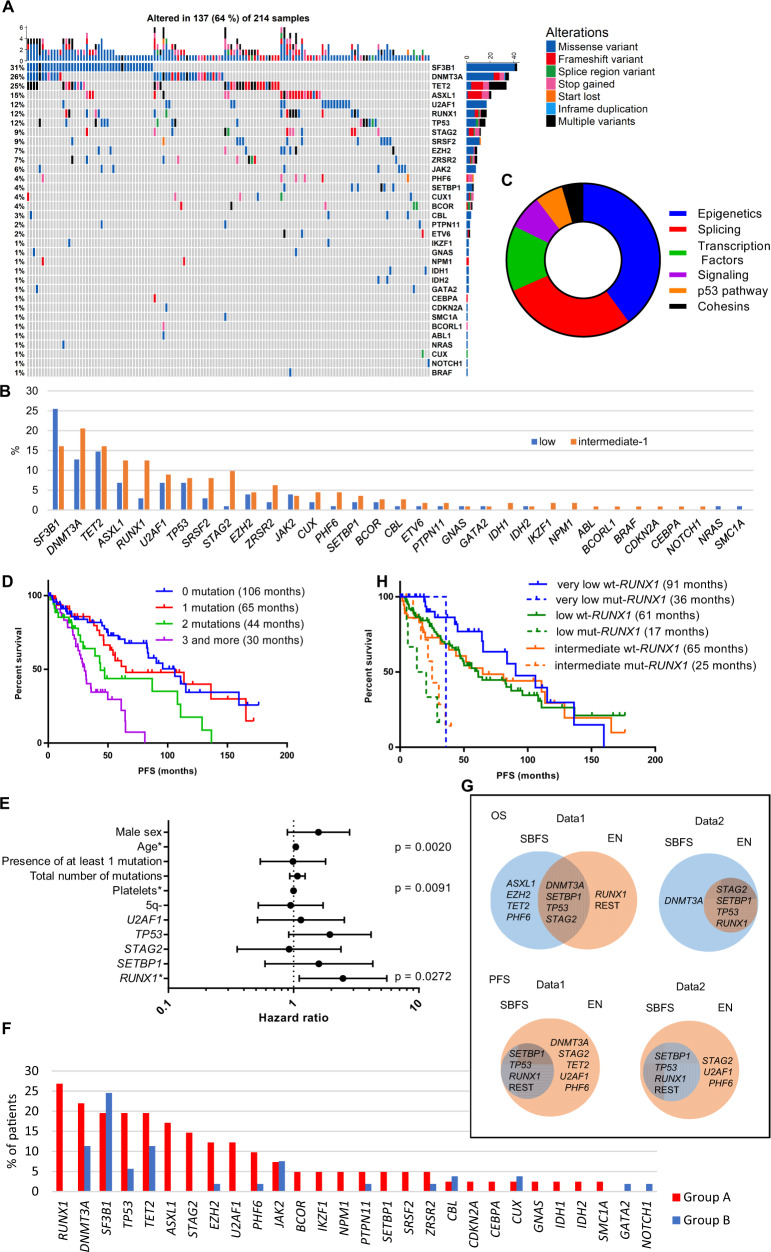
The landscape of mutated genes in the cohort of 214 LR-MDS patients. **A** Distribution, cooccurrence, and type of mutations in 137 of 214 LR-MDS patients. Each column represents an individual sample. The colored cells indicate a mutation in the gene described in the row on the right. The color indicates the type of alteration. The percentage on the left indicates the representation of mutated genes in 137 patients with mutations. The upper columns illustrate the number of mutations in the samples. The right stripes demonstrate the number of mutations of the gene throughout our cohort. **B** The most frequently mutated genes grouped by low and intermediate-1 IPSS risk groups. The Y-axis indicates the percent representation in the cohort. **C** Mutated genes grouped by functional categories. The most represented categories were epigenetic regulators (blue) and splicing regulators (red). **D** Effect of the number of mutations on PFS, *p* < 0.0001, with the median PFS in parentheses. **E** Multivariate analysis of mutational and clinical variables that were significant in univariate analysis of PFS depicted in a forest plot (hazard ratio, confidence intervals). Details are listed in SI 5C. * indicates significant independent prognostic factors. **F** The mutational landscape at the time of diagnosis in two groups of patients according to their progression within 5 years. Group A included patients who progressed within 5 years, and group B included patients who did not progress and were followed for at least 5 years. **G** Results of both machine learning methods (multivariate Cox regression with stepwise backward feature selection (SBFS) and elastic networks (EN)) applied to OS and PFS in datasets 1 (data1: binary mutational data) and 2 (data2: the number of distinct mutations per gene) depicted in Venn diagrams. The results of SBFS are depicted in blue circles, and the results of EN are depicted in orange circles. Common results are shown in overlaps. **H** Kaplan–Meier survival curves of patients stratified by IPSS-R and mutational status of the *RUNX1* gene, *p* < 0.0001, with the median OS in parentheses. wt-*RUNX1*, patients without *RUNX1* mutations, mut-*RUNX1*, patients with *RUNX1* mutations.

We characterized the mutational landscape of 54 tested genes in the LR-MDS patient cohort at diagnosis (Fig. 1A). At least one pathogenic mutation was found in 137 patients (64%); in greater detail, pathogenic mutations were found in 53% of low-risk patients and 74% of INT-1. The number of mutations ranged from 0 to 9. The mutational complexity of co-occurrences is depicted in a Circos plot (SI 4A). The most common mutated gene was *SF3B1*, which was identified in 21% of patients, followed by *DNMT3A* in 17% of patients. The mutational profiles of the low-risk group and INT-1 group are depicted in Fig. 1B. In terms of functional categories, the most frequently mutated genes were epigenetic regulators (42%) (Fig. 1C) classified according to Sperling, Gibson, & Ebert [21].

Univariate analyses for overall survival (OS) and progression-free survival (PFS) (time from diagnosis until progression or death) were performed for BM blast count, cytopenias, IPSS and IPSS-R score, male sex, age, and presence of a 5q deletion and mutated genes (detected in more than five patients) (SI 5A). The significant variables in both analyses (*p* < 0.05) were platelet count, male sex, age, and the presence and total number of mutations. Significantly mutated genes for OS were *DNMT3A*, *RUNX1*, *SETBP1*, *STAG2*, and *TP53*, while mutated *RUNX1*, *SETBP1*, *STAG2*, *TP53*, and *U2AF1* were significant for PFS. OS and PFS decreased as the number of mutations increased (Fig. 1D). The presence of the deletion of 5q was significant for PFS and, in contrast to other variables, increased PFS. Neither IPSS nor IPSS-R showed significant differences between groups in our cohort (SI 6). However, adding information on the mutational status of genes that were significant in the univariate analysis led to great diversification of the OS and PFS curves among the groups (SI 7). Platelet count, age, and mutated *TP53* and *DNMT3A* were the most significant variables for OS in multivariate analysis of all significant variables from the univariate analysis (SI 5B). Considering a recent report on the effect of allelic status of *TP53* mutations on MDS prognosis [22], out of 16 patients carrying *TP53* mutations, 11 seemed to carry a monoallelic mutation. However, we could consider the allelic status only according to the number of identified mutations and their VAF. The median VAF of *TP53* mutations at diagnosis was 10% (range, 1–52%). Platelet count, age, and mutated *RUNX1* were the most significant independent prognostic factors in the multivariate analysis for PFS (Figs. 1E, SI 5C). Thus, the effect of *RUNX1* mutations on shortened PFS indicates its potential significance as a marker of rapid progression. Detailed statistical data are available in Supplement (SI 5).

### The mutational landscape is different between patients with and without rapid progression

We compared the baseline characteristics of patients who progressed within 5 years (group A) to those without progression (or who progressed later than 5 years) (group B). We censored the patients who were not monitored for at least 5 years and patients who underwent HSCT up to 5 years from diagnosis. Therefore, 41 patients who progressed rapidly (group A) and 53 patients who did not progress (group B) were compared. The median time to progression in group A was 19.8 months.

Between these groups, significant differences were observed in the median age (*p* = 0.0030), male sex (*p* = 0.0197), and platelet count (*p* = 0.0003). The median OS was 33 months for group A and 136 months for group B (*p* < 0.0001) (SI 8). More detailed information on the patients is described in SI 9.

Eighty-five percent of the patients in group A and 47% of the patients in group B carried at least one mutation. The median number of mutations in group A was 3 (range 0–8), while it was 0 (range 0–5) in group B. The landscape of mutated genes was very different between the groups (Fig. 1F). The most commonly mutated gene in group A was *RUNX1* (27%); in contrast, this gene was not mutated in group B at all. The most commonly mutated gene in group B was *SF3B1* (25%), and this gene was mutated in 20% of patients in group A. Highly mutated genes in group A that were wild-type in group B included *ASXL1*, *STAG2*, and *U2AF1*.

### The mutational burden is higher during progression

We compared the mutational landscapes of paired samples from 36 patients who progressed within 5 years (before vs. after progression). We identified 24 new mutations in samples after progression. The greatest increase in the total number of mutations (114%) was observed in genes involved in signaling pathways (SI 10). Generally, the VAF of mutations increased from diagnosis to progression with few exceptions. Examples of VAF changes in paired samples are shown in SI 11.

### Machine learning applied to mutational data confirms the significant effect of mutations on survival

According to the multivariate Cox regression with stepwise backward feature selection (SBFS), the mutated gene responsible for the shortest OS was *STAG2* in dataset 1 (binary mutational data) and *RUNX1* in dataset 2 (the number of distinct mutations per gene) (SI 12A). For the shortest PFS, *RUNX1* was mutated in both datasets.

According to the cross-validation experiments for SBFS and elastic network (EN) models (Supplementary Results), the optimal number of genes responsible for a shorter OS and PFS was greater than 1. The most significant genes are listed in Tables SI 12B and SI 13B for the individual datasets. Both methods identified mutated *DNMT3A*, *SETBP1*, *TP53*, and *STAG2* as significant for OS in dataset 1 and mutated *STAG2*, *SETBP1*, *TP53*, and *RUNX1* as significant for dataset 2. In both datasets, significant genes for shorter PFS identified by both methods were *SETBP1*, *TP53*, and *RUNX1*. The complex results from both methods are depicted in Figs. 1G and SI 14.

When the SBFS model was extended with comutational data (SI 15A-B), the presence of mutated *RUNX1* and *EZH2* together had the strongest impact on OS and PFS. In the EN approach, including gene interactions in the model did not improve its quality.

Because the initial number of independent variables was too large with respect to the number of events, the full models led to overfitting. The regularized models with smaller feature sets outperformed the full models. At the same time, they were significantly better than random, which confirms our hypothesis that risk stratification in MDS may be improved by including molecular data. The predicted hazards for the individual subjects could be used to assume their survival (described in the Supplementary Results).

### *RUNX1* mutational status can improve risk stratification of LR-MDS

We identified 25 unique mutations in *RUNX1* in 17 patients at diagnosis and in 2 patients who developed *RUNX1* mutations during progression (SI 16). Eighteen of the identified *RUNX1* mutations (75%) were located in the Runt homology domain (RUNT), which is responsible for DNA binding and interaction with CBFβ (SI 16). Overall, most mutations remove residues that are important for RUNX1 activity, suggesting a loss of RUNX1 function in these mutants [23]. Some mutations are likely dominant-negative [18], and in some mutants, the effect could not be predicted without functional assays [24]. All *RUNX1* mutations were proven to be somatic (except for one presented in a patient whose CD3 + cells were not available). Most mutations were present at a lower VAF (<10%). *ASXL1, EZH2*, and *STAG2* were most frequently comutated with *RUNX1* (SI 4B).

*RUNX1* mutational status significantly affected the IPSS-R scores (Fig. 1H). After adding information on *RUNX1* mutational status to the IPSS-R scoring system, the survival curves divided patients into two groups: i) patients with prolonged PFS from the three risk classes without any *RUNX1* mutation (wt-*RUNX1*) and ii) patients with shortened PFS with *RUNX1* mutations (mut-*RUNX1*).

Comparing the clinical features between *RUNX1*-mutated patients and others in our cohort revealed significant differences in the BM blast and platelet counts and the median number of mutations (SI 17).

### The antitumor cellular response is downregulated in *RUNX1*-mutated LR-MDS

Because mutated *RUNX1* showed the greatest impact on rapid progression, we aimed to analyze the mechanism by which mutations in this gene contribute to rapid progression. We compared the transcriptomes of CD34 + cells between 8 *RUNX1-*mutated lower-risk patients (mutR-LR) and 29 lower-risk patients without *RUNX1* mutations (wtR-LR) (SI 3).

**Fig. 2:**
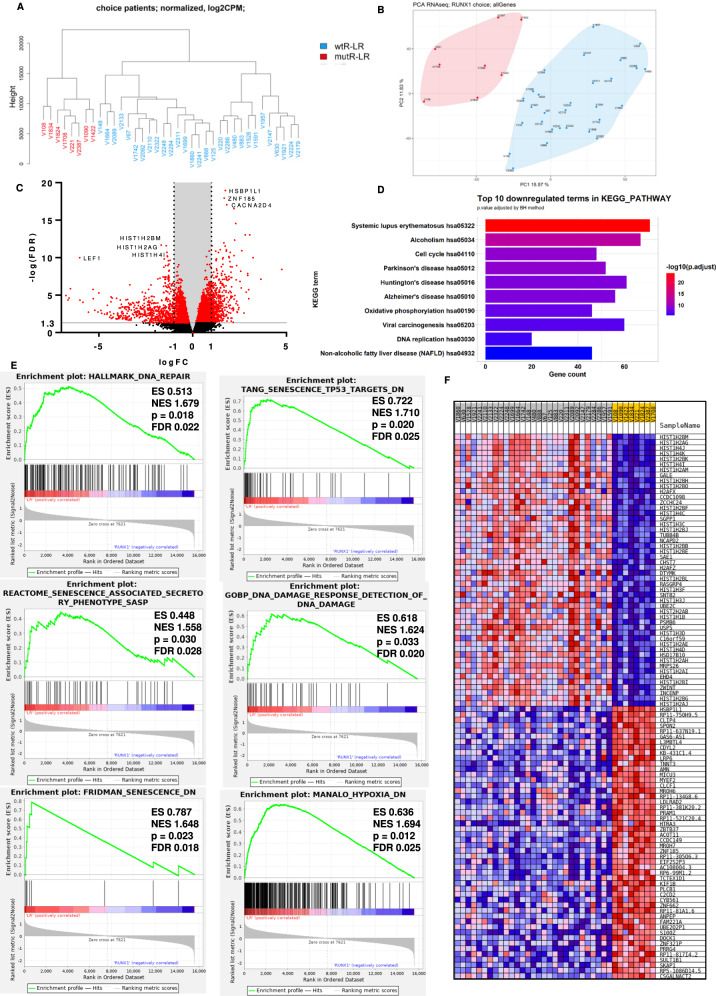
Transcriptome analysis of mutR-LR and wtR-LR RNA-seq data. **A** Hierarchical clustering and **B** PCA of *RUNX1*-mutated (mutR-LR) and *RUNX1*-unmutated LR-MDS patients (wtR-LR). **C** Differentially expressed genes depicted in a volcano plot. The red points indicate significantly dysregulated genes between CD34 + cells from lower-risk MDS patients with and without *RUNX1* mutations. *x*-axis: logFC, logarithm of fold-changes; *y*-axis: −log10 of FDR value; FDR - false discovery rate, red points: FDR < 0.05. **D** Top 10 downregulated KEGG pathways in mutR-LR compared to wtR-LR by *p* value. *x*-axis: number of genes in the pathway; color depicts adjusted *p* value (the highest values are red). **E** Six of 82 significantly (FDR < 0.25) dysregulated pathways by GSEA in the custom dataset consisting of 88 gene sets linked to the DNA repair, DNA damage response, cellular senescence, apoptosis, and hypoxia pathways. ES, enrichment score; NES, normalized enrichment score; p, *p* value; FDR, false discovery rate. **F** Heatmap representing the expression profiles of the top 50 up- and down-regulated genes in the custom dataset. mutR-LR highlighted in yellow, wtR-LR highlighted in gray. Gene expression levels are represented by colors; red represents upregulated genes and blue represents downregulated genes. The intensity indicates the level of differential expression.

Hierarchical clustering (Fig. 2A) and principal component analysis (Fig. 2B) of RNA-seq data showed differences in the expression profiles of mutR-LR from those of wtR-LR. In the differential expression analysis of mutR-LR versus wtR-LR, 2235 genes were significantly (FDR < 0.05) upregulated and 2094 were significantly downregulated (Fig. 2C). Differentially expressed genes were enriched in 641 GO biological processes. GO enrichment analysis (GOrilla) [25] reduced this number to 103. The main pathways that had significant FDR values were chromatin and gene silencing, nucleosome assembly, chromatin organization, regulation of megakaryocyte differentiation and myeloid cell differentiation and hemopoiesis, telomere organization and capping, cellular metabolic processes, DNA damage response (DDR) and DNA repair, and cellular response to stress. The top 10 up- and down-regulated terms in GO biological processes are visualized in the Supplementary Material (SI 18A, B).

In the KEGG database, 47 pathways were significantly enriched. The top 10 upregulated KEGG pathways in mutR-LR were related to cancer and leukemia (SI 18C). The top 10 downregulated KEGG pathways were pathways of neurodegenerative diseases, inflammatory response, and cell cycle (Fig. 2D). These pathways are tightly connected to DDR and DNA repair, cellular senescence, aging, chronic inflammation, oxidative stress, and apoptosis [26–30], which all play a role in cellular tumor protection.

In our custom dataset consisting of 88 gene sets connected to DDR, DNA repair, cellular senescence, apoptosis, and hypoxia, 82 gene sets were significantly enriched in wtR-LR (FDR < 0.1). Enrichment plots and the heatmap of the top 50 genes are depicted in Fig. 2E, F.

**Fig. 3:**
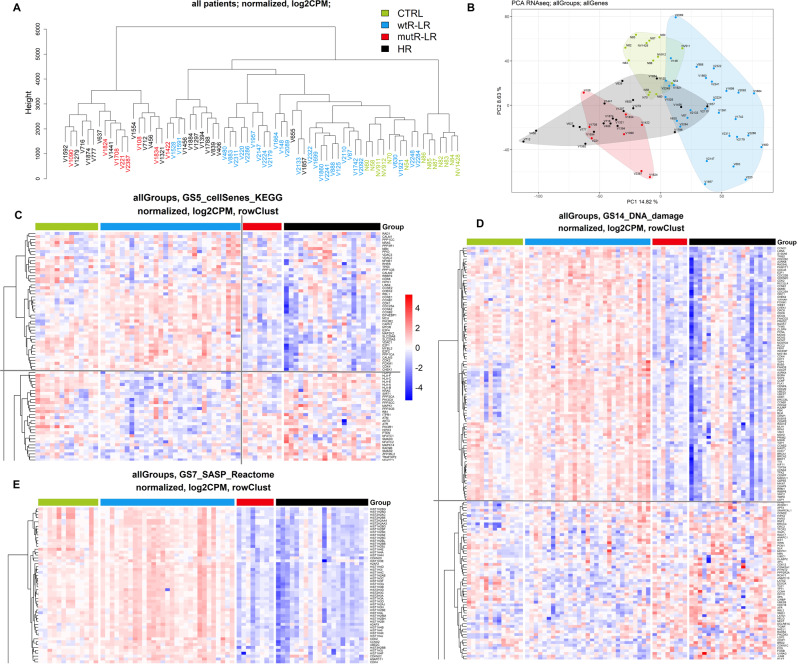
Transcriptome analysis of LR- and HR-MDS patients. **A** Hierarchical clustering and **B** PCA of CD34 + cells of mutR-LR, wtR-LR, HR and healthy controls (CTRL). Heatmaps displaying significantly dysregulated expression in selected genes of GSEA pathways: **C** Cellular senescence (KEGG), **D** DNA damage, **E** SASP (Reactome). Red indicates upregulation, blue indicates downregulation of gene expression, and the color intensity indicates the level of differential expression. The heatmaps including all genes of the pathways are shown in SI 20.

To better understand the differences between mutR-LR and wtR-LR, we supplemented the cohort with 20 higher-risk patients (HR) and 13 healthy controls (median age 41 years) (SI 3) and compared the expression profiles of CD34 + cells. Interestingly, mutR-LR patients clustered with HR patients (Fig. 3A, B). In dysregulated GSEA pathways, mutR-LR CD34 + cells transcriptionally resembled HR cells, indicating transcriptional similarity with HR patient cells already at diagnosis (Fig. 3C–E).

### Markers of senescence are dysregulated in *RUNX1*-mutated LR-MDS and HR-MDS cells

**Fig. 4:**
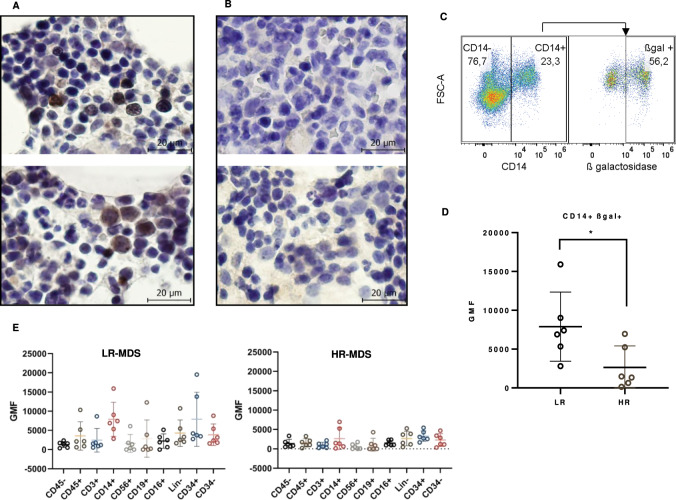
Detection of markers of cellular senescence. Immunohistochemical staining of γH2AX protein in BM FFPE sections of **A** wtR-LR patients and **B** mutR-LR patients. Images of the two wtR-LR patients show highly positive zones of the sections that were not present in the mutR-LR patients. **C** Representative example of the gating strategy of CD14 + cells and SA-β-gal expression. The numbers in the plot indicate the percentage of gated cells. **D** Significant difference in activity (GMF) of SA-β-gal between LR- and HR-MDS CD14 + cells (median, interquartile range), *p* = 0.026, calculated using two-sided Mann-Whitney test. **E** Geometric mean fluorescence (GMF) represents the level of SA-β-gal activity for LR-MDS and HR-MDS patients in immune cell subsets from bone marrow (median, interquartile range).

To validate the suppression of DDR and senescence in cells of LR-MDS patients with *RUNX1* mutations and HR-MDS patients compared to that in cells of LR-MDS patients without *RUNX1* mutations, we performed two types of analysis: i) immunohistochemical staining of γH2AX on BM FFPE sections and ii) fluorescence detection of senescence-associated β-galactosidase (SA-β-gal) activity in BM sorted cells. We observed higher staining of γH2AX in *RUNX1*-unmutated samples than in *RUNX1*-mutated samples, where the marker was very low or undetectable (Fig. 4A, B, SI 19). Furthermore, significantly higher SA-β-gal activity, indicating a higher percentage of senescent cells, was observed in CD14 + monocytes of LR-MDS compared to HR-MDS (Fig. 4C, D). The CD34 + cell results had to be omitted in statistical analyses because of the low number of CD34 + cells in samples, and mutR-LR samples were not available for this assay. However, based on the expression profiles of senescence-associated pathways in mutR-LR and HR-MDS (Fig. 3C–E; SI 20), similar results for SA-β-gal can be anticipated in mutR-LR. Generally, the detected fluorescence levels among cell types were much more uniform in HR-MDS samples than in LR-MDS (Fig. 4E). A gating strategy example is depicted in SI 21.

## Discussion

This study aimed to describe the MDS mutational landscape using NGS technology, which is unique for a cohort composed exclusively of LR-MDS patients. To our knowledge, the only study exclusively targeting LR-MDS patients and aiming to enhance the prognostic system with molecular data thus far was published in 2012 and consisted of 288 LR-MDS patients [9]; however, few genes were sequenced, and the prognosis was based only on OS, not PFS. In this context, our study describes a novel, unique strategy for MDS stratification based on molecular markers and machine learning methods.

In our cohort, at least one pathogenic mutation was detected in 64% of patients. One of the most frequently mutated genes was *SF3B1*, which is in line with other studies [11, 12, 31]. This gene did not have a significant effect on OS, as reported earlier; however, these previous studies evaluated the effect in the entire spectrum of patients with MDS from low- to high-risk. Because *SF3B1* is predominantly mutated in lower-risk patients, its effect on survival may appear greater in the unstratified MDS cohort than in the lower-risk cohort.

Incorporation of the mutational status of genes affecting OS or PFS into IPSS-R significantly improved risk stratification. In multivariate analysis, age, platelet count, mutated *TP53* and *DNMT3A* were significant for OS, and age, platelets, and mutated *RUNX1* were significant for PFS. We have previously reported platelet count as well as mutated *TP53* as one of the strongest independent prognostic factors for OS in LR-MDS [32]. Unfavorable outcomes related to *RUNX1* mutations were described in a 16-study meta-analysis of MDS patients without risk stratification [33].

Machine learning is an emerging approach for risk stratification in various disorders, including MDS [13, 34, 35]. Nevertheless, to date, no algorithm has been used globally to stratify patients or predict the disease course. In our cohort, machine learning showed that mutated *RUNX1*, *TP53,* and *SETBP1* are significant predictors of rapid progression, with *RUNX1* being the main factor.

Due to the strong effect of mutated *RUNX1* on PFS, we further aimed to investigate this gene and its role in progression. According to the VAF of *RUNX1* mutations and other commutated genes in *RUNX1*-mutated patients, we suppose that *RUNX1* mutations are not founder mutations but rather subsequent events in clonal evolution contributing to cell transformation. Similar conclusions were drawn by earlier studies [12, 36].

To determine the dysregulated molecular pathways associated with mutated *RUNX1*, we compared the expression profiles of CD34 + cells of LR-MDS patients with (mutR-LR) and without (wtR-LR) *RUNX1* mutations. Overall, data from differential expression analysis and GSEA showed suppression of pathways associated with antitumor cellular response—DDR, cellular senescence, chromatin and gene silencing, apoptosis, cellular response to stress, telomere maintenance, and hypoxia—in mutR-LR patients.

These data indicate the role of *RUNX1* as a tumor suppressor in LR-MDS and suggest a functional impact of *RUNX1* mutations, direct or indirect, in eliminating a biological anticancer barrier against accelerated progression in LR-MDS patients. We found that wtR-LR CD34 + cells activate the DDR and attain hallmarks of senescence, resulting in delayed progression. Indeed, senescence has been described as a part of the tumorigenesis barrier in premalignant lesions [37–39]. With the assumption that DDR and senescence are activated in the vicinity of senescent cells by senescence-associated secretory phenotype (SASP) [40, 41], we measured SA-β-gal expression in several BM sorted cell types and showed its significantly higher level, particularly in CD14 + monocytes of wtR-LR-MDS. Our transcriptional comparison of SASP genes also suggests that senescence-associated inflammatory cytokine secretion (as described by Rodier et al. [42]) serves as a local microenvironmental mediator of the LR-MDS cellular state, contributing to the barrier against malignant progression and enforcing DDR activation, a phenomenon we proposed to be a barrier counteracting the progression of preleukemia to leukemia [43]. Our data also suggest that while some wtR-LR BM progenitors activate the DDR (marked by γH2AX), including increased DNA repair capacity consistent with proliferation, some wtR-LR BM cells suffer more DNA damage and undergo senescence. Thus, wt*RUNX1* is functionally intertwined with DDR in LR-MDS in our cohort, and *RUNX1* mutations are associated with elimination of the DDR-mediated senescence barrier and accelerated disease progression.

Several studies have shown that wt*RUNX1* contributes to the protection of cells against oncogenesis. It is necessary for the p53 response to DNA damage [44], and knockdown of this gene may cause escape from senescence and enhance apoptosis suppression [45]. *RUNX1* also interacts with a subunit of *HIF1*, *HIF-1ɒ*, and inhibits its transcriptional activity [46]. Overexpression of *HIF-1ɒ* may result in tumor angiogenesis and tumor progression [47].

In our cohort, *HIF1* and hypoxia cellular response pathways were significantly dysregulated in mutR-LR, which may impact the origin of senescence [48]. *HIF1* and hypoxia are known to have an antisenescent effect [48–50]; however, they can induce the transcription of SASP genes and thus promote senescence in a paracrine fashion [48]. The dysregulation of *HIF1* and hypoxia cellular response pathways has been described in various types of tumors [47, 51, 52].

Our data also show that mutR-LR cell expression profiles are more similar to those of HR-MDS cells than to those of wtR-LR cells at the time of diagnosis. We previously demonstrated that CD34 + cells of patients with early MDS show significant overexpression of genes involved in the cell cycle, DDR and DNA repair compared to those from advanced MDS patients [53]. Suppression of the DDR in AML cells versus MDS cells [54] and downregulation of homologous recombination gene expression in high-risk compared to low-risk MDS patients [55] have been reported. Similarly, a decrease in the expression of DNA damage checkpoints and dysregulation of the cell cycle were described in advanced MDS [56].

To conclude, this study shows that MDS risk stratification may be improved by including molecular data. Based on these data, we can identify patients at risk of rapid progression and choose proper follow-up and treatment strategies. LR-MDS patients with a *RUNX1* mutation at diagnosis should be intensively monitored despite the lower-risk group. Transcriptome data suggest that *RUNX1* mutations disrupt the fail-safe mechanism in hematopoietic stem cells and contribute to rapid progression in LR-MDS.

## Supplementary information

Supplementary Material

List of Mutations

DEA

## Data Availability

Raw data were deposited in the National Center for Biotechnology Information (NCBI) Sequence Read Archive (SRA) database (accession number PRJNA797993).
