# Supplementary material for: RUNX1 mutations contribute to the progression of MDS due to disruption of antitumor cellular defense: a study on patients with lower-risk MDS
Source: Leukemia. 2022 May 3;36(7):1898–906. doi: 10.1038/s41375-022-01584-3 (PMC9252911; doi:10.1038/s41375-022-01584-3)
Supplement: Supplementary file 1 — Supplementary Material [file 41375_2022_1584_MOESM1_ESM.pdf]

***RUNX1* mutations contribute to the progression of MDS due to disruption of antitumor cellular defense: A study on patients with lower-risk MDS**

Supplementary Material

1. **Supplementary Methods**
2. **Supplementary Results**
3. **Supplementary Figures and Tables**

1. SUPPLEMENTARY METHODS

DNA and RNA Isolation

For the preparation of the DNA library, DNA from bone marrow (BM) or, if BM was not available, peripheral blood (PB) was isolated using MagCore according to the manufacturers´ recommendations (RBC Bioscience, New Taipei City, Taiwan). DNA concentration was measured by Qubit 3.0 fluorometer (Life Technologies, Carlsbad, CA, USA) and quality was checked using Nanodrop (Thermo Fisher Scientific, Waltham, MA, USA).

For the preparation of the RNA library, BM CD34+ cells were isolated by magnetic separation on an autoMACS Separator (Miltenyi Biotec, Bergisch Gladbach, Germany). Then, RNA was isolated by acid guanidinium thiocyanate-phenol-chloroform extraction. RNA concentration was measured with a Qubit 3.0 fluorometer (Life Technologies) and RNA quality was checked on an Agilent 2100 bioanalyzer (Agilent Technologies, Palo Alto, CA, USA). Only high-quality RNA with an RNA integrity number (RIN) of at least 7.5 was used.

Targeted gene sequencing

50 ng of DNA were used to prepare the indexed library according to the manufacturer's protocol (TruSight Myeloid Sequencing Panel Kit, Illumina, San Diego, CA, USA). Quantification of the prepared library was done with the KAPA Library Quantification Kit for Illumina sequencing platforms (KAPA Biosystems, Wilmington, MA, USA) also according to the manufacturer's protocol. Sequencing was performed on the MiSeq or NextSeq (Illumina). Analysis was performed with NextGene software (SoftGenetics, State College, PA, USA) and in-house pipeline (described separately in the following paragraph). In paired samples, variants with VAF up to 0.01 were detected if the variant was present in VAF more than 0.05 in one of the paired samples. SIFT and Polyphen were used for the prediction of variant effects. Visualization of NGS results was done with R 4.0.2 software (circlize 0.4.13, ComplexHeatmap 2.8.0).

In-house pipeline for analyzing NGS data from TruSight Myeloid Sequencing Panel:

The quality of raw data obtained from high-throughput sequencing was checked by FastQC version 0.11.8. Reads were then trimmed and filtered using Trimmomatic software version 0.39 and resulting files were quality checked by FastQC again. Cleaned up data from DNA sequencing were then mapped to GRCh19 version of human genome using BWA aligner version 0.7.17. The mapped data was further indexed and sorted using the Samtools suite of tools version 1.10 and the percentage of mapped reads was assessed. Samples with a percentage of mapped reads exceeding 95 % were processed using variant calling software Freebayes version 1.3.1. To discover additional insertions and deletions that span across long sections of the genome, Pindel software version 0.2.5b9 was used. The discovered variants in the form of VCF files were then filtered and annotated using the online interface of Ensembl Variant Effect Predictor (VEP). The annotated variants were formatted in the R software version 4.0.2 and then exported to TSV format.

Sanger sequencing

DNA from CD3+ cells was used in PCR reaction with Q5 Hot Start High-Fidelity DNA Polymerase (New England Biolabs, Ipswich, MA, USA) and the PCR reaction was run according to the manufacturer's protocol. The sequences of the primers are in Table SI 1. 10 µl of the PCR product was run on the 1% TAE gel at 80 V to control the PCR reaction. The PCR product was then cleaned with ExaSap-IT PCR Product Cleanup Reagent (Thermo Fisher Scientific) according to the manufacturer's protocol. The next step was to prepare the sequencing PCR reaction with the BigDye Terminator v3.1 Cycle Sequencing

Kit (Thermo Fisher Scientific) using 0.5 μl of the PCR product. The PCR products were then cleaned with the DyeEx 2.0 Spin Kit (QIAGEN, Venlo, The Netherlands) according to the manufacturer's protocol. Finally, the sample was analyzed on the ABI 3500 (Thermo Fisher Scientific) and the sequences were visualized on Sequencing Analysis Software 5.4 (Thermo Fisher Scientific).

RNA sequencing

100 ng of total RNA from CD34+ cells of 8 LR-MDS patients with *RUNX1* mutation, 29 LR-MDS without *RUNX1* mutation, 20 HR-MDS and 13 healthy controls (SI 3) was ribodepleted with the RiboCop rRNA Depletion Kit (Lexogen, Wien, Austria). Sequencing was performed on HiSeq 2500 or NovaSeq (Illumina). Raw reads in the form of FASTQ files were trimmed and filtered using Trimmomatic 0.39 and their quality was assessed using FastQC 0.11.8. The quantification of gene expression was performed by StringTie2 software 1.3.6. The filtered reads were mapped to human genome GRCh38.p13 using STAR 2.7.2b. The quantification of gene expression was performed by StringTie2 software 1.3.6. For analysis and visualization of expression data, several packages in R software 4.0.2 (e.g. edgeR 3.30.3, pheatmap 1.0.12, ggplot 3.3.2, pcaMethods 1.84.0, ComplexHeatmap 2.8.0) and GraphPad Prism 7 software (GraphPad Software, La Jolla, CA, USA) were used. Databases such as Gene Ontology, KEGG Pathways, Reactome Pathways, and ConsensusPathDB were used for functional enrichment analysis.

Machine Learning

Genes with negligible mutation occurrence (mutated in fewer than 6 subjects) were grouped into one category (REST). The variables were therefore: *ASXL1*, *CUX1*, *DNMT3A*, *EZH2*, *JAK2*, *PHF6*, *RUNX1*, *SETBP1*, *SF3B1*, *SRSF2*, *STAG2*, *TET2*, *TP53*, *U2AF1*, *ZRSR2*, REST. In multivariate Cox regression with stepwise backward feature selection, the Aikake information criterion was used by default (rms R library, fastbw function). It is a heuristic criterion, and its application led to very small models of only one gene. Then, after the cross-validated experiment, the model was adjusted for the optimal number of features according to the D value and the general recommendations for the number of events per variable (EPV) in survival regression models (Peduzzi et al. 1995). In our data, we had 214 subjects, 81 death events; then the EPV should be around 10, which means we should have not worked with more than 8 features. Lasso regression (elastic networks) that worked with L1 norm and minimized the number of features was used. The optimal parameterization/number of features was set in cross-validation again, the model quality was measured with the Harrell's C-index (the concordance index, its value is between 0 and 1). We got two recommendations, lambda.min (optimum) and lambda.1se (a regularized model near optimum result).

β-galactosidase detection

Cocktail of antibodies: CD3 – Spark Blue 550 (Biolegend, San Diego, CA, USA), CD14 – Alexa Fluor 594 (Biolegend), CD16 – BV650 (Biolegend), CD19 – BV570 (Biolegend), CD34 – eFluor 450 (eBioscience, San Diego, CA, USA), CD45 – Alexa Fluor 647 (Biolegend), CD56 – APC fire 750 (Biolegend), LIVE/DEAD fixable blue dead cell stain kit (Invitrogen, Carlsbad, CA, USA).

2. SUPPLEMENTARY RESULTS

Machine Learning – Cross-Validation

In the cross-validation experiments of SBFS, the maximum D-value was approximately 0.17 for data1 and 0.18 for data2 in OS, respectively. The maximum D-value for both PFS datasets was approximately 0.25. In Table SI 11B, the most significant genes are listed in the number that should be ideal for individual datasets according to the cross-validated value of the D measure. Extending the model with comutational data, the maximal D value increased in OS but decreased in PFS (SI 14C).

In cross-validating EN, the highest C-index was around 0.6 in all analyses (SI 12A) and the number of optimal features is specified within the table of results (SI 12B). Comutational data did not improve the C-index.

Machine Learning – Individual hazard ratio model

We used our data to create a hazard ratio model to count the hazard ratio for individual patients.

The predictions corresponded to logarithmic relative hazards:

$\log(h_k(t)/h_{k'}(t))= \log(h_0(t)^{(\beta 1x1k+ \beta 2x2k+\ldots + \beta pxpk)}/ \log(h_0(t)^{(\beta 1x1k'+ \beta 2x2k'+\ldots + \beta pxpk')})= \beta_1(x_{1k}- x_{1k'}) + \beta_2(x_{2k}- x_{2k'})+\ldots + \beta_p(x_{pk}- x_{pk'})$, where x_i denoted the ith covariate (mutations in our case), β_i the ith coefficient (the effect size of the given covariate) and the individual k' represented the baseline (average) individual. Counting the hazard ratios between individuals, $h_0(t)$ became unimportant since it remained the same for all the individuals. Therefore, we were able to count the relative hazard ratio for individual patients from individual analysis.

3. SUPPLEMENTARY FIGURES AND TABLES

PATIENT CHARACTERISTICS AT THE TIME OF DIAGNOSIS, all patients	
**Number of patients**	214
**Age median (years) (range)**	65 (20.8-86.5)
**Sex**	
**Male**	107 (50.0%)
**Female**	107 (50.0%)
**Laboratory data**	**Median (range)**
**BM blasts (%)**	2 (0-9.8)
**Haemoglobin (g/dL)**	10 (5.1-14.9)
**ANC (10^9/L)**	2 (0.1-9.2)
**Platelets (10^9/L)**	195 (1.0-1115.0)
**Cytogenetics (IPSS)**	
**Good**	188 (87.9%)
**Intermediate**	22 (10.3%)
**Poor**	4 (1.9%)
**IPSS**	
**Low**	102 (47.7%)
**Intermediate I**	112 (52.3%)
**Intermediate II**	0
**High**	0
**IPSS - R**	
**Very low**	45 (21.0%)
**Low**	119 (55.6%)
**Intermediate**	46 (21.5%)
**High**	4 (1.9%)
**Very high**	0
**WHO classification (2016)**	
**MDS-MLD**	113 (52,8%)
**MDS-SLD**	20 (9,3%)
**MDS-del(5q)**	37 (17,3%)
**MDS-RS**	22 (10,3%)
**MDS-EB-1**	18 (8,4%)
**MDS-EB-2**	3 (1,4%)
**MDS-U**	1 (0,5%)

SI 1: Patient characteristics at the time of diagnosis. BM, bone marrow; ANC, absolute neutrophil count.

Primers for exons 5-7	Sequence 5'-3'	Ta
RUNX1_5F	TCCCTGATGTCTGCATTTGTCC	66
RUNX1_5R	AGACAGACCGAGTTTCTAGGG	
RUNX1_6F	AGCAAAGCCAAAATTCCGGG	67
RUNX1_6R	GGTCCCTGAGTATACCAGCCT	
RUNX1_7F	AGCGAGTCTATGTTGGGGTG	68
RUNX1_7R	AAGGGGAAACCCCAGTTGGT	

SI 2. Primers for PCR and sequencing variants in exons 5-7 of the *RUNX1* gene. Ta, annealing temperature; F, forward primer; R, reverse primer.

Sample	Diagnosis (WHO)	Cyto-genetics (IPSS)	Mutations (VAF %) in total BM DNA	*VAF (%)* of *RUNX1* mutations in cDNA from CD34+
RUNX1-LR-MDS				
V108	MDS-EB-1	good	*RUNX1* (35 and 3 and 4), *SF3B1* (36), *TET2* (9), *IKZF1* (10)	61; 12; 4
V1834	MDS-EB-1	good	*RUNX1* (35), *SF3B1* (37), *EZH2* (7)	46
V1824	MDS-EB-2	good	*RUNX1* (11), *SF3B1* (42), *ASXL1* (25), *ZRSR2* (25), *STAG2* (35)	31
V1708	MDS-EB-2	good	*RUNX1* (44), *SRSF2* (40), *STAG2* (89), *ASXL1* (43)	42
V221	MDS-EB-1	good	*RUNX1* (10), *SETBP1* (4), *STAG2* (12)	23
V2387	MDS-EB-1	good	*RUNX1* (14 and 12), *ASXL1* (30), *STAG2* (14)	17; 19
V1090	MDS-EB-1	good	*RUNX1* (41 and 2), *ASXL1* (21), *GNAS* (50), *PHF6* (43), *EZH2* (39 and 40)	15; 43
V1422	MDS-MLD	good	*RUNX1* (49), *SRSF2* (51), *SETBP1* (48)	53
LR-MDS				
V148	MDS-MLD	good	*U2AF1* (43), *TET2* (3)	
V1664	MDS-MLD	Int	*DNMT3A* (11 and 12)	
V2089	MDS-SLD	good	*U2AF1* (40)	
V2133	MDS-MLD	good	none	
V67	MDS-RS	good	*SF3B1* (46), *DNMT3A* (46)	
V1742	MDS-RS	good	*SF3B1* (48), *DNMT3A* (50), *TET2* (42 and 37), *CUX1* (5)	
V2092	MDS-RS	good	*SF3B1* (29), *TET2* (21)	
V2110	MDS-MLD	good	none	
V2322	MDS-MLD	good	none	
V2248	MDS-MLD	good	none	
V2284	MDS-MLD	good	*U2AF1* (14)	
V2311	MDS-MLD	good	*TP53* (34)	
V1699	MDS-RS	good	*SF3B1* (40), *TET2* (34)	
V1860	MDS-RS	good	none	
V2241	MDS-SLD	good	none	
V888	MDS-RS	good	*SF3B1* (28 and 4)	
V125	MDS-SLD	good	*SF3B1* (27)	
V220	MDS-MLD	int	*SF3B1* (43)	
V2286	MDS-MLD	good	*SF3B1* (38)	
V480	MDS-del(5q)	good	*DNMT3A* (24)	
V883	MDS-RS	good	*SF3B1* (42), *TET2* (19)	
V1528	MDS-del(5q)	good	none	
V1591	MDS-MLD	good	*SF3B1* (10)	
V1957	MDS-EB-1	good	*ASXL1* (29); *PHF6* (82)	
V2147	MDS-MLD	good	*TET2* (30 and 40)	
V630	MDS-MLD	good	none	
V1921	MDS-SLD	good	none	
V2224	MDS-SLD	good	none	
V2179	MDS-SLD	good	*TET2* (32)	
HR-MDS				
V1592	MDS-EB-2	int	*TET2* (45), *RUNX1* (45), *ASXL1* (50), *EZH2* (49), *PHF6* (45)	
V1279	MDS-EB-2	good	*TET2* (19 and 22), *EZH2* (6), *ZRSR2* (68)	
V716	MDS-EB-2	poor	*SF3B1* (18)	
V1874	AML-MRC	N/A	none	
V777	AML-MRC	good	none	
V637	MDS-EB-2	int	none	
V1441	MDS-EB-2	good	*RUNX1* (30), *TET2* (6), *BCOR* (28)	
V1554	AML -MRC	good	*IDH2* (5), *IKZF1* (22), *STAG2* (6)	
V712	MDS-EB-2	good	*SRSF2* (32), *ASXL1* (26), *RUNX1* (30), *BCOR* (6), *STAG2* (6 and 45)	
V456	MDS-EB-2	good	none	
V1321	MDS-EB-1	good	*SF3B1* (9)	
V1456	MDS-MLD	int	*SF3B1* (39), *TET2* (46), *CUX1* (49)	
V1884	MDS-EB-2	N/A	*TP53* (36 and 40)	
V1297	MDS-EB-2	poor	*TP53* (10)	
V1394	MDS-EB-2	N/A	*NRAS* (46), *ETV6* (49), *ASXL1* (38), *STAG2* (90), *PHF6* (96), *GATA2* (43)	
V1788	MDS-EB-2	poor	*TP53* (59), *SF3B1* (40)	
V839	MDS-EB-2	Int	*DNMT3A* (44), *RUNX1* (26)	
V406	MDS-EB-2	int	*SF3B1* (27)	
V655	MDS-EB-2	poor	*TP53* (69)	
V1857	MDS-EB-2	good	*DNMT3A* (41)	

SI 3. The list of patients in the expression study with their cytogenetic and mutation profiles. Thirteen healthy controls are not included. VAF, variant allele frequency; BM, bone marrow; R-LR, *RUNX1*-mutated LR-MDS

patients; wtR-LR – LR-MDS without *RUNX1* mutations; HR, higher-risk MDS patients; int, intermediate; N/A, not available.

A

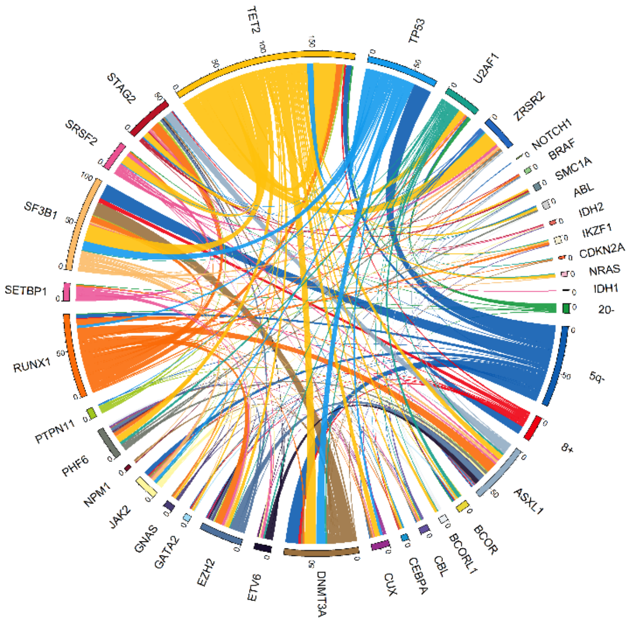

B

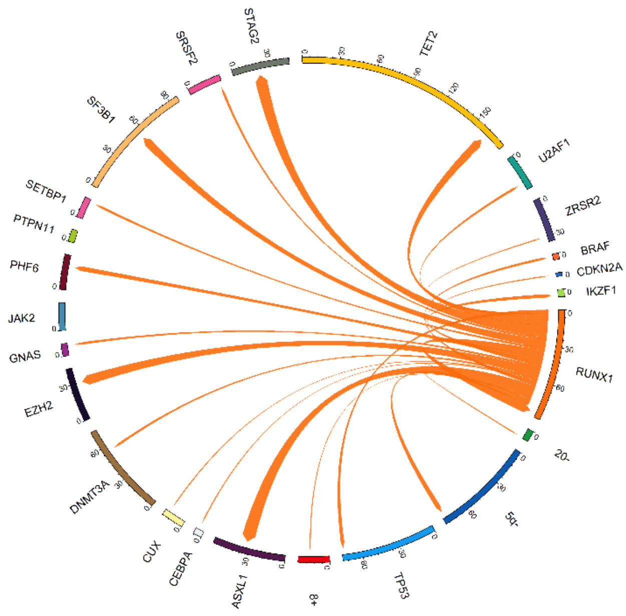

SI 4. Circos plots illustrating pairwise co-occurrences of selected genetic alterations. A) Circos plot of all pairwise co-occurrences identified in the Czech cohort of 214 LR-MDS patients. B) Circos plot of the co-occurrence of molecular aberrations with the mutated *RUNX1* gene. The length of the arch depicts the number of mutations of the first gene comutated with other mutations. The width of the ribbon corresponds to the frequency of co-occurence with the second gene.

A

Univariate analysis	p value	
Variable	OS	PFS
ASXL1	ns	ns
CUX	ns	ns
DNMT3A	0.0286	ns
EZH2	ns	ns
JAK2	ns	ns
PHF6	ns	ns
RUNX1	0.0005	<0.0001
SETBP1	0.0201	0.0225
SF3B1	ns	ns
SRSF2	ns	ns
STAG2	0.0004	0.0019
TET2	ns	ns
TP53	0.0154	0.0487
U2AF1	ns	0.0426
ZRSR2	ns	ns
Male sex	0.0003	<0.0001
Presence of at least 1 mutation	0.0071	0.0016
IPSS	ns	ns
IPSS-R	ns	ns
BM blasts	ns	ns
Platelet counts	0.0015	0.0004
Haemoglobin	ns	ns
ANC	ns	ns
Total number of mutations	0.0001	<0.0001
5q-	ns	0.0348
Age	<0.0001	<0.0001

B

Variable - OS	p value univariate	p value multivariate	HR	95% CI of HR
Male sex	0.0003	0.3125	1.0832	0.9276 to 1.2648
Age	<0.0001	<0.0001	1.0605	1.0344 to 1.0872
Presence of at least 1 mutation	0.0071	0.3926	0.7405	0.3719 to 1.4745
Total number of mutations	0.0001	0.0039	0.9971	0.9951 to 0.9991
Platelet counts	0.0015	0.4022	1.2776	0.7202 to 2.2662
TP53	0.0154	0.0405	2.0931	1.0326 to 4.2424
STAG2	0.0004	0.6950	1.1980	0.4855 to 2.9564
SETBP1	0.0201	0.0649	2.8633	0.9371 to 8.7490
RUNX1	0.0005	0.2680	1.6272	0.6876 to 3.8509
DNMT3A	0.0286	0.0492	1.8803	1.0022 to 3.5280

C

Variable - PFS	p value univariate	p value multivariate	HR	95% CI of HR
Male sex	<0.0001	0.1164	1.5805	0.8926 to 2.7989
Age	<0.0001	0.0002	1.0404	1.0187 to 1.0626
Presence of at least 1 mutation	0.0016	0.9679	0.9877	0.5419 to 1.8002
Total number of mutations	<0.0001	0.3303	1.0724	0.9316 to 1.2343
Platelet counts	0.0004	0.0091	0.9977	0.9959 to 0.9994
5q-	0.0348	0.8576	0.9470	0.5224 to 1.7167
U2AF1	0.0426	0.7427	1.1430	0.5146 to 2.5384
TP53	0.0487	0.0849	1.9462	0.9124 to 4.1516
STAG2	0.0019	0.8639	0.9200	0.3544 to 2.3877
SETBP1	0.0225	0.3584	1.5904	0.5908 to 4.2812
RUNX1	<0.0001	0.0272	2.4782	1.1077 to 5.5443

SI 5. Univariate and multivariate analyses. A) The tested variables and p values for OS and PFS in univariate analysis. Only genes mutated in more than 5 patients were tested. All significant variables of the univariate analysis ($p < 0.05$) were analysed in the multivariate analysis: B) OS, C) PFS. The significant variables in the multivariate analysis ($p < 0.05$) are highlighted. ns, not significant; BM, bone marrow; ANC, absolute neutrophil count; HR, hazard ratio; CI, confidence intervals of the hazard ratios.

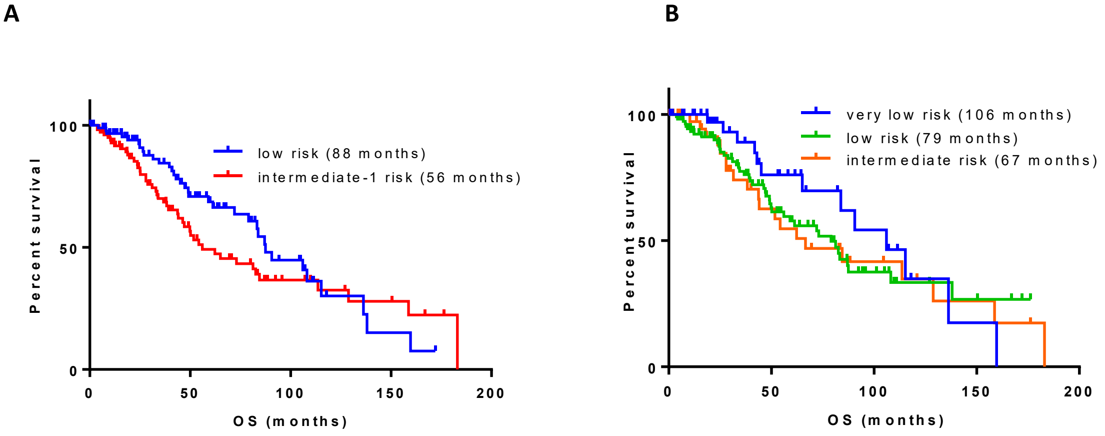

SI 6. Overall survival (OS) of patients according to their IPSS (A) and IPSS-R (B) scores. Neither was significant. Median OS in parentheses.

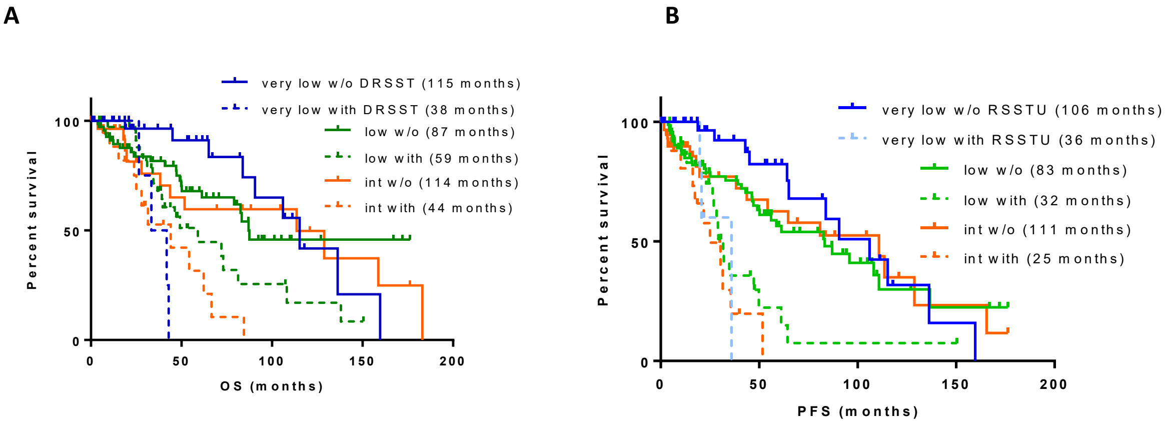

SI 7. Integration of the mutational status of significant genes in univariate analysis into the IPSS-R system. A) The graph shows overall survival curves of patients with or without at least one mutated gene of *DNMT3A*, *RUNX1*, *SETBP1*, *STAG2*, and *TP53* (DRSST), $p < 0.0001$. B) Implementation of the mutational status of *RUNX1*, *SETBP1*, *STAG2*, *TP53*, and *U2AF1* (RSSTU), $p < 0.0001$. Patients with RSSTU mutations are those with mutations in at least one of these genes. Intermediate, int; without, w/o. Median OS/PFS is in parentheses.

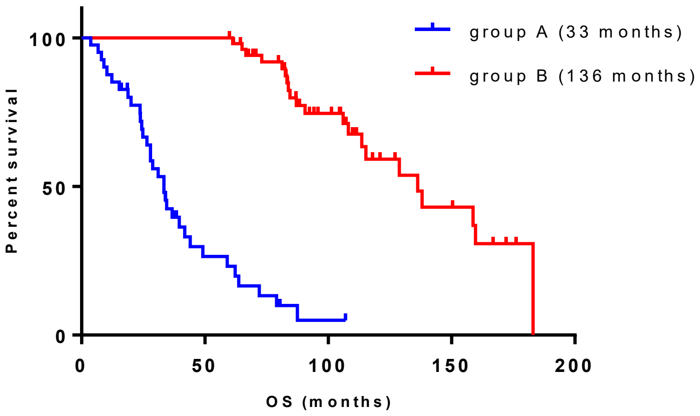

SI 8. OS curves for group A (progressed within 5 years) and group B (did not progress within 5 years), $p < 0.0001$, median OS in parentheses.

PATIENT CHARACTERISTICS AT THE TIME OF DIAGNOSIS (who progressed within 5 years or were followed at least 5 years)			
	**Group A**	**Group B**	**P value**
**Number of patients**	41	53	-
**Age median* (years) (range)**	68 (28.2-86.5)	58 (20.8-84.4)	0.003
**Sex***			0.0197
**Male**	23 (56.1%)	16 (30.2%)	-
**Female**	18 (43.9%)	37 (69.8%)	-
**Laboratory data**	**Median (range)**		
**BM blasts (%)**	4 (0.4-8.6)	2 (0-7.6)	ns
**Hemoglobin (g/dL)**	10 (7.5-14.6)	10 (6.1-13.6)	ns
**ANC (10^9/L)**	2 (0.1-7.5)	2 (0.4-6.9)	ns
**Platelets* (10^9/L)**	150 (15-406)	284 (25-1115)	0.0003
**Cytogenetics (IPSS)**			
**Good**	37 (90.2%)	48 (90.6%)	ns
**Intermediate**	4 (9.8%)	5 (9.4%)	ns
**Poor**	0	0	-
**IPSS**			
**Low**	15 (36.6%)	26 (49.1%)	ns
**Intermediate I**	26 (63.4%)	27 (50.9%)	ns
**Intermediate II**	0	0	-
**High**	0	0	-
**IPSS-R**			
**Very low**	4 (9.8%)	13 (24.5%)	ns
**Low**	26 (63.4%)	27 (51.0%)	ns
**Intermediate**	10 (24.4%)	13 (24.5%)	ns
**High**	1 (2.4%)	0	-
**Very high**	0	0	-
**WHO classification (2016)**			
**MDS-MLD**	21 (51,2%)	19 (35.9%)	ns
**MDS-SLD**	2 (4,9%)	3 (5.7%)	ns
**MDS-del(5q)**	10 (24.4%)	20 (37.7%)	ns
**MDS-RS**	2 (4,9%)	7 (13.2%)	ns
**MDS-EB-1**	6 (14.6%)	4 (7.5%)	ns
**MDS-EB-2**	0	0	-
**MDS-U**	0	0	-

SI 9: Patient characteristics at the time of diagnosis for two groups of patients. Group A – patients who progressed within 5 years, group B – patients who did not progress within 5 years, but were followed at least 5 years. * indicates values significantly different between groups A and B; ns, not significant; BM, bone marrow; ANC, absolute neutrophil count.

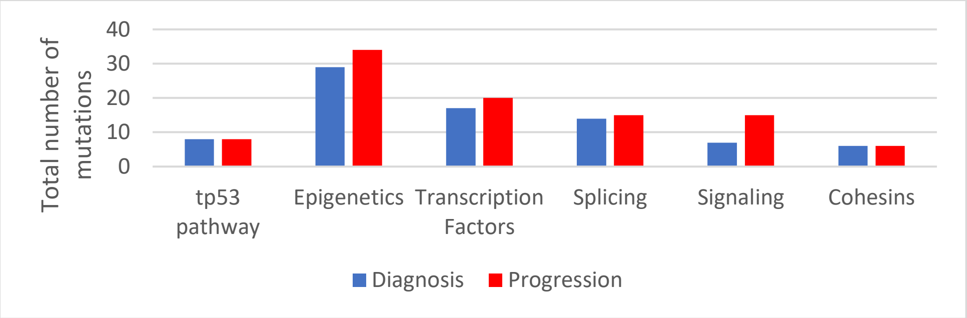

SI 10: Change in the number of mutations by functional categories in paired samples from the time of diagnosis and progression. The blue columns represent the total number of mutations at diagnosis, and the red columns represent the progression.

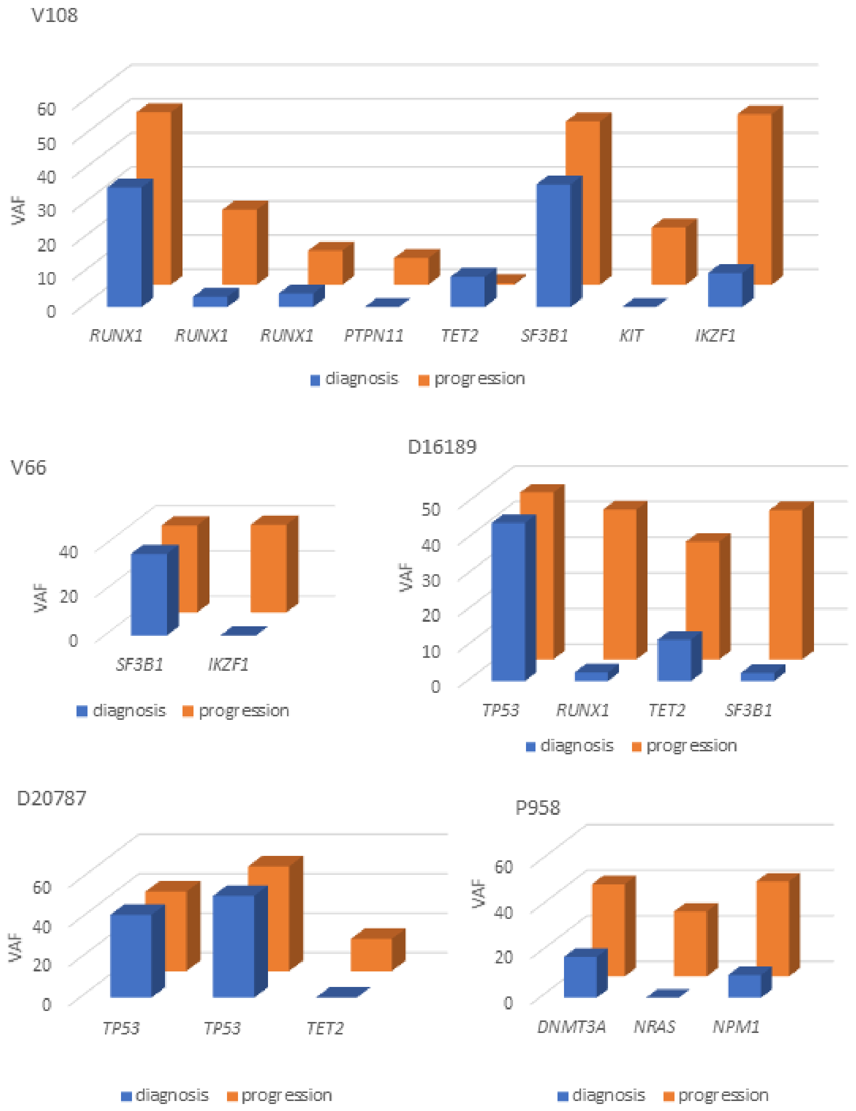

SI 11: Progression-related changes in VAF of individual samples. Examples of 5 patients sequenced at the time of diagnosis (blue) and in progression (orange). In general, there was an increase in VAF from diagnosis to progression, but not exclusively. Very often, a novel mutation was found in progression. VAF – variant allele frequency (%).

A

	Coef	S.E.	Wald Z	Pr(>|Z|)
OS_data1:				
STAG2	1.356	0.4172	3.25	0.001156
OS_data2:				
RUNX1	0.9267	0.2518	3.679	0.0002338
PFS_data1:				
RUNX1	1.4463	0.3301	4.38	<0.0001
PFS_data2:				
RUNX1	1.0109	0.2153	4.70	<0.0001

B

	Coef	S.E.	Wald Z	Pr(>|Z|)
OS_data1:				
ASXL1	0.5253	0.4823	1.09	0.2761
EZH2	-0.8193	0.7089	-1.16	0.2478
TET2	0.4467	0.3035	1.47	0.1412
PHF6	1.0501	0.5309	1.98	0.0479
DNMT3A	0.5144	0.2793	1.84	0.0655
SETBP1	1.6311	0.5795	2.81	0.0049
TP53	0.7984	0.3387	2.36	0.0184
STAG2	0.9723	0.4770	2.04	0.0415
OS_data2:				
DNMT3A	0.3508	0.2178	1.61	0.1073
STAG2	0.6609	0.3664	1.80	0.0713
SETBP1	0.9309	0.4075	2.28	0.0223
TP53	0.3935	0.1802	2.18	0.0290
RUNX1	0.7447	0.2700	2.76	0.0058
PFS_data1				
SETBP1	0.5529	0.4885	1.13	0.2578
TP53	0.5459	0.3296	1.66	0.0977
REST	0.8023	0.2610	3.07	0.0021
RUNX1	1.2577	0.3458	3.64	0.0003
PFS_data2				
SETBP1	0.6228	0.3835	1.62	0.1043
TP53	0.4227	0.1783	2.37	0.0177
REST	0.4304	0.1948	2.21	0.0271
RUNX1	0.9529	0.2198	4.33	<0.0001

SI 12. The results of the stepwise backward feature selection for both datasets of OS and PFS. A) Genes responsible for the shortest OS. B) Optimal number of features responsible for shorter OS and PFS according to cross-validated D-value. Data1, dataset 1, binary mutational data; data2, dataset 2, the number of distinct mutations per gene; coef, coefficient; S.E., standard error; Wald z; Wald test z value; Pr(>|Z|), p value.

A

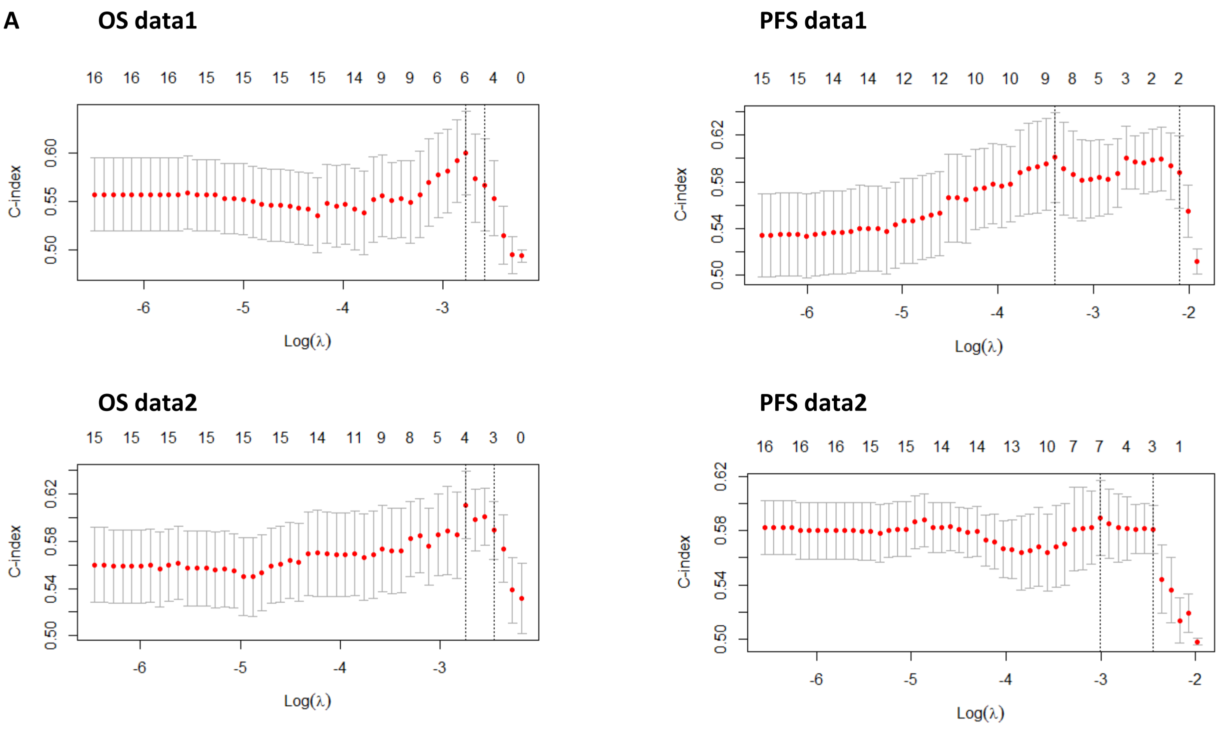

B

	Coef lambda.min	Coef lambda.1se
OS_data1 (6 genes with lambda.min, 5 genes with lambda.1se), C-index 0.60:		
DNMT3A	0.1546945	0.06630357
RUNX1	0.4350070	0.36033025
SETBP1	0.1535746	.
STAG2	0.4307018	0.31244418
TP53	0.2333416	0.13650722
REST	0.1072556	0.01689362
OS_data2 (4 genes with lambda.min, 3 genes with lambda.1se), C-index 0.61:		
RUNX1	0.4317455	0.31356625
SETBP1	0.2150566	.
STAG2	0.2770572	0.08685572
TP53	0.2113882	0.11858889
PFS_data1 (9 genes with lambda.min, 2 genes with lambda.1se), C-index 0.6:		
DNMT3A	0.1523	.
PHF6	0.0054	.
RUNX1	1.0552	0.6183
SETBP1	0.3186	.
STAG2	0.1374	.
TET2	0.0498	.
TP53	0.3448	.
U2AF1	0.1662	.
REST	0.5519	0.2047
PFS_data2 (7 genes with lambda.min, 3 genes with lambda.1se), C-index 0.59:		
PHF6	0.0506	.
RUNX1	0.7080	0.5405
SETBP1	0.2790	.
STAG2	0.1228	.
TP53	0.2429	0.0619
U2AF1	0.0968	.
REST	0.2152	0.0670

SI 13. OS and PFS analysis by elastic network approach. A) Cross-validating plots indicating the number of features with the highest C-index for both datasets of OS and PFS. B) Results indicating the most significant genes responsible for shorter OS and PFS. Data1, dataset 1, binary mutational data; data2, dataset 2, the number of distinct mutations per gene; lambda.min, the minimum mean cross-validated error; lambda.1se, the value when the cross-validated error is within one standard error of the minimum, that gives the most regularized model.

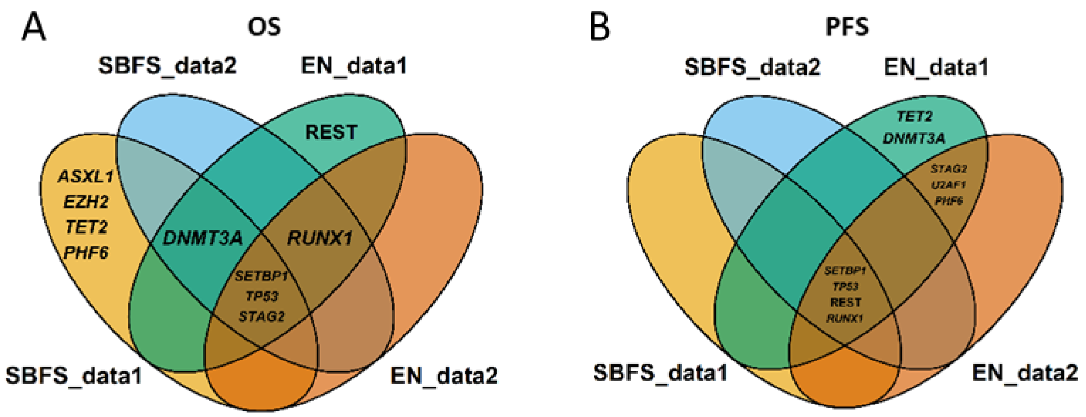

SI 14: Venn diagrams depicting the results of different machine learning methods. Multivariate Cox regression with stepwise backward feature selection and elastic network methods were used on two datasets for A) OS as well as B) PFS. SBFS, multivariate Cox regression with stepwise backward feature selection; EN, elastic networks; data1, dataset 1, binary mutational data; data2, dataset 2, the number of distinct mutations per gene.

A

Interaction	Freq	Fisher exact test p-value
DNMT3A-SF3B1	12	4.415016e-02
SF3B1-TET2	12	1.981671e-02
ASXL1-RUNX1	8	3.054957e-05
ASXL1-STAG2	8	8.356220e-07
RUNX1-STAG2	7	3.355163e-06
ASXL1-EZH2	5	5.515366e-04
TET2-ZRSR2	5	5.218261e-03
EZH2-RUNX1	4	2.735664e-03
SRSF2-TET2	4	9.402812e-02

B

	Coef	S.E.	Wald z	Pr(>|Z|)
OS_data1				
ASXL1	0.7190	0.5148	1.40	0.1625
EZH2	-1.9953	1.1960	-1.67	0.0953
ZRSR2	-1.2313	0.8451	-1.46	0.1451
SRSF2	-0.0308	0.7844	-0.04	0.9687
TET2	0.4733	0.3218	1.47	0.1413
DNMT3A	0.5335	0.2852	1.87	0.0614
SETBP1	1.8196	0.8119	2.24	0.0250
TP53	0.7193	0.3488	2.06	0.0392
RUNX1	0.0983	0.5046	0.19	0.8456
STAG2	0.9471	0.5325	1.78	0.0753
*SRSF2*TET2*	1.7668	1.4002	1.26	0.2070
*EZH2*RUNX1*	3.0166	1.5151	1.99	0.0465
PFS_data1				
ASXL1	0.7760	0.5915	1.31	0.1896
RUNX1	1.3547	0.5172	2.62	0.0088
TP53	0.4570	0.3584	1.28	0.2022
EZH2	-0.3875	0.9551	-0.41	0.6850
SETBP1	0.8947	0.6326	1.41	0.1573
U2AF1	0.8584	0.4356	1.97	0.0487
DNMT3A	0.5790	0.2715	2.13	0.0330
TET2	0.1363	0.3145	0.43	0.6646
ZRSR2	-1.0543	1.0765	-0.98	0.3274
STAG2	1.4398	0.6655	2.16	0.0305
*ASXL1*RUNX1*	-1.4470	1.0875	-1.33	0.1833
*ASXL1*EZH2*	-1.2378	1.2145	-1.02	0.3081
*RUNX1*EZH2*	2.6793	1.2394	2.16	0.0306
*TET2*ZRSR2*	2.3201	1.2924	1.80	0.0726
*RUNX1*STAG2*	-1.3304	1.0084	-1.32	0.1870

C

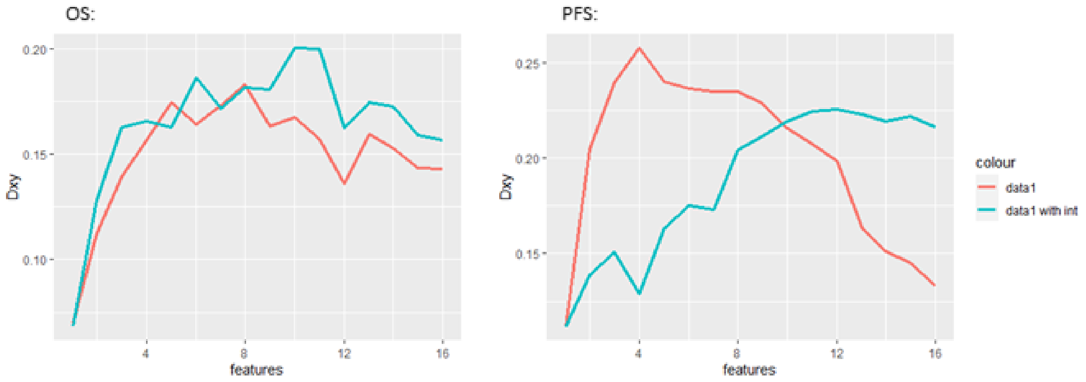

SI 15. Effect of interaction between comutated genes on survival. A) Significant (p<0.05) interactions between comutated genes for dataset 1 according to their effect on survival. Interactions with the REST category were omitted, as well as interactions with frequency less than 4. Freq, frequency of interactions in the cohort. B) Multivariate Cox regression with stepwise backward feature selection (SBFS) model counting with the effect of single mutations and comutations. C) Cross-validation plots of the D value depicting the difference between the SBFS model with and without interactions for OS and PFS. Data1, dataset 1, binary mutational data; coef, coefficient; S.E., standard error; Wald z; Wald test z value; Pr(>|Z|), p value.

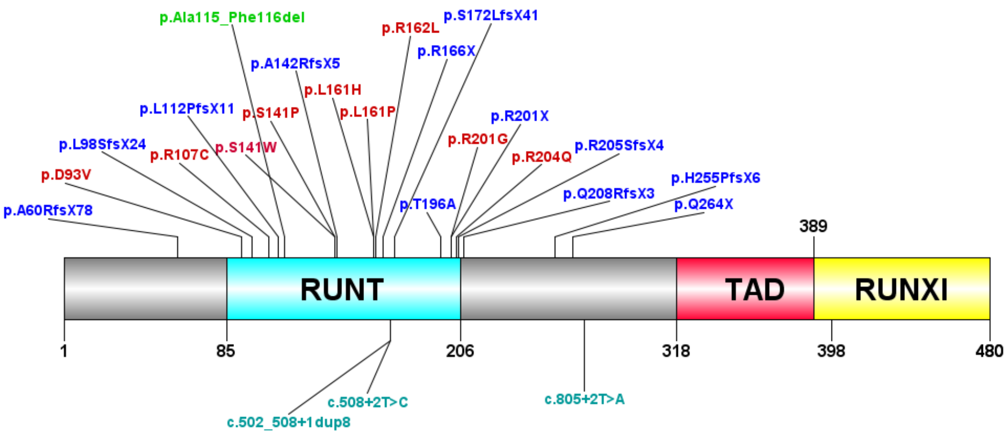

SI 16. Schematic illustration of the *RUNX1* gene and the mutations identified in the cohort of 214 Czech LR-MDS patients. Most mutations lied in the Runt homology domain (RUNT); no mutation was located in two others domains - the transcriptional activation domain (TAD) and the Runx1 inhibition domain (RUNXI). The illustration was created using DOG 2.0 (Yao & Xue, 2009). The mutations are distinguished by colors: red – missense, blue – frameshift and stop gain, green – in-frame deletion, teal – splice region.

PATIENT CHARACTERISTICS AT THE TIME OF DIAGNOSIS			
	RUNX1 mutated	*RUNX1* wildtype	p value
**Number of patients**	17	197	-
**Age median (years) (range)**	66 (28-78)	64 (21-87)	ns
**Sex**			
**Male**	10 (58.8%)	97 (49.2%)	ns
**Female**	7 (41.2%)	100 (50.8%)	
**Laboratory data**	**Median (range)**		
**BM blasts* (%)**	5.0 (0.8-9.8)	1.8 (0.0-8.8)	<0,001
**Hemoglobin (g/dL)**	9.5 (7.8-12.8)	9.8 (5.1-14.9)	ns
**ANC (10**^{9}/L)	1.6 (0.6-7.5)	1.8 (0.1-9.2)	ns
**Platelets* (10**^{9}/L)	103 (15-313)	202.5 (1-1115)	0.010
**Cytogenetics (IPSS)**			
**Good**	16 (94.1%)	172 (87.3%)	ns
**Intermediate**	1 (5.9%)	21 (10.7%)	
**Poor**	0	4 (2.0%)	
**IPSS***			
**Low**	3 (17.6%)	99 (50.3%)	0.011
**Intermediate I**	14 (82.4%)	98 (49.7%)	
**Intermediate II**	0	0	-
**High**	0	0	-
**IPSS-R***			
**Very low**	1 (5.9%)	44 (22.3%)	0.004
**Low**	6 (35.3%)	113 (57.4%)	
**Intermediate**	9 (52.9%)	37 (18.8%)	
**High**	1 (5.9%)	3 (1.5%)	
**Very high**	0	0	-
**WHO classification (2016)**			
**MDS-MLD**	5 (29.4%)	108 (54.8%)	<0,001
**MDS-SLD**	0	20 (10.2%)	
**MDS-del(5q)**	2 (11.8%)	35 (17.8%)	
**MDS-RS**	1 (5.9%)	21 (10.7%)	
**MDS-EB-1**	7 (41.2%)	11 (5.6%)	
**MDS-EB-2**	2 (11.8%)	1 (0.5%)	
**MDS-U**	0	1 (0.5%)	
**Mutation data**			
**No. of mutations**	4 (2-7)	1 (0-9)	<0,001

SI 17. Baseline characteristics of lower-risk MDS patients with and without *RUNX1* mutations. BM, bone marrow; ANC, absolute neutrophil count; ns, not significant; * indicates significantly different values between groups.

A

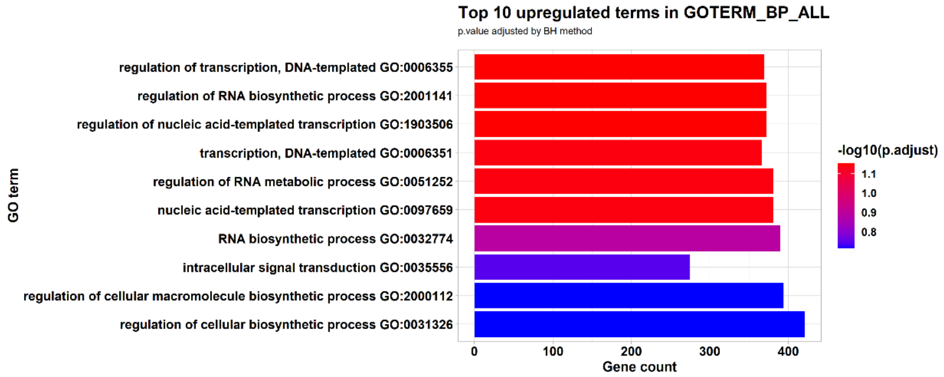

B

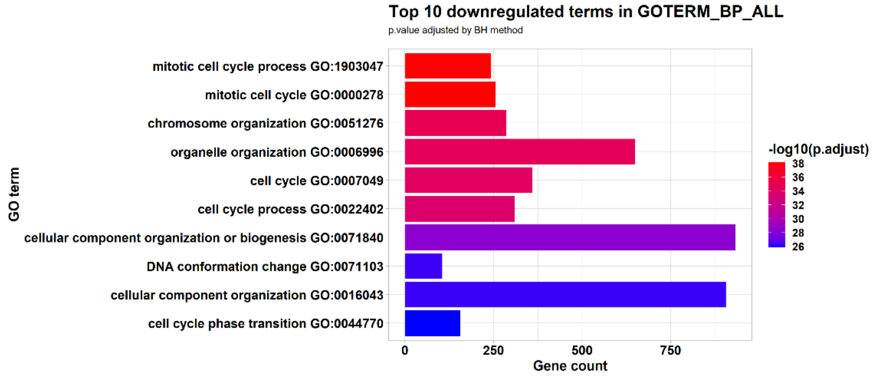

C

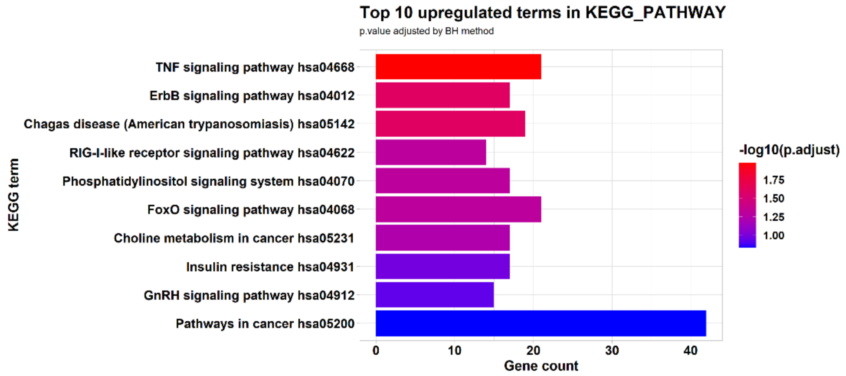

SI 18. Top 10 up- and downregulated terms in GO biological processes (A-B) and top 10 upregulated KEGG pathways (C) for mutR-LR compared to wtR-LR by p value. Colors indicate the level of significance.

% of positive cells	4 wtR-LR	3 mutR-LR
Minimum	0	0
25% Percentile	0.25	0
Median	6.5	0
75% Percentile	12.75	2
Maximum	13	2
Mean	6.5	0.6667
Std. Deviation	6.952	1.155
Std. Error of Mean	3.476	0.6667
Lower 95% CI of mean	-4.563	-2.202
Upper 95% CI of mean	17.56	3.535

SI 19: Quantification of the percentage of cells expressing γH2AX in 4 wtR-LR and 3 mutR-LR patients. The γH2AX staining was evaluated in three to five fields of view of individual samples representing different zones of stained sections.

A

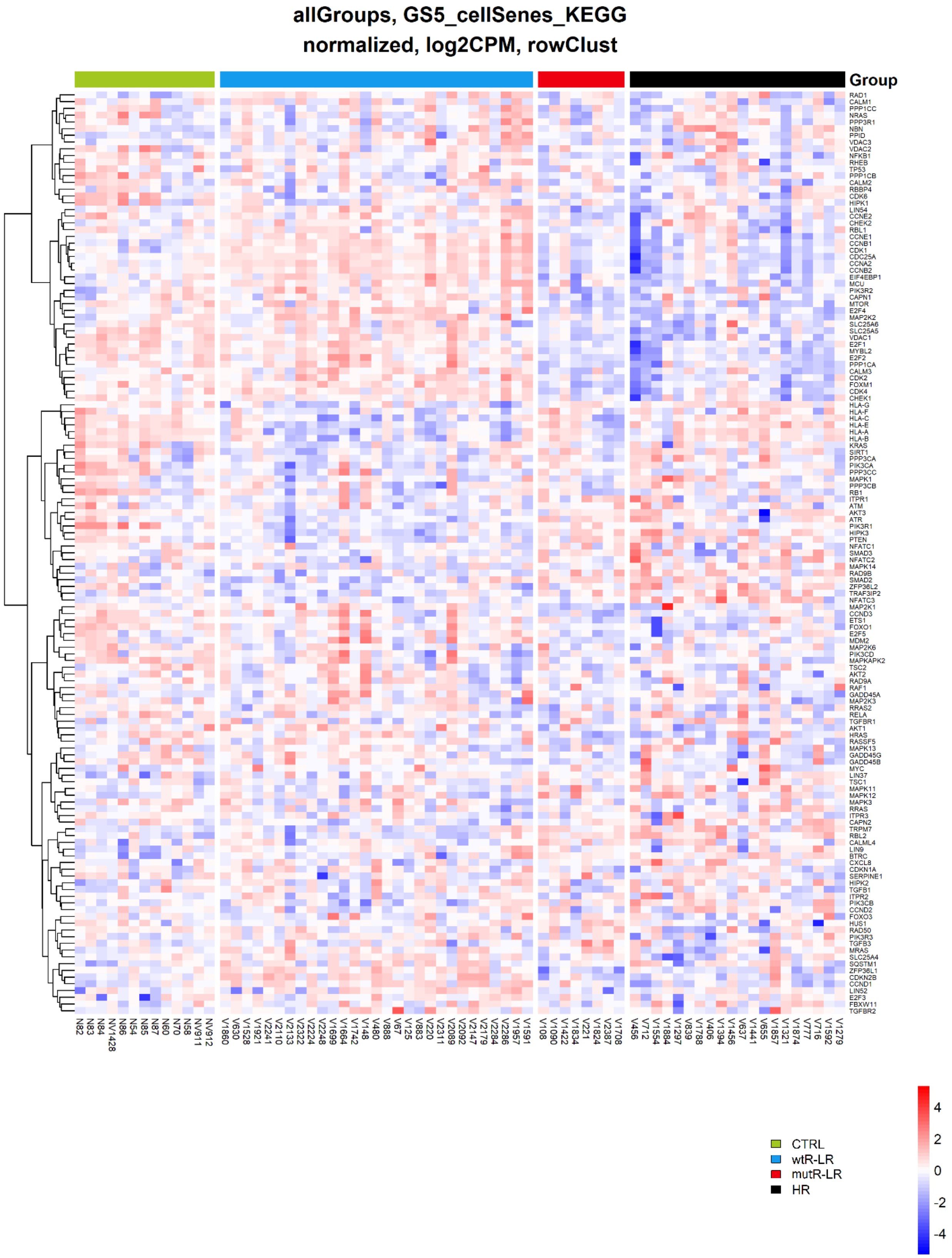
allGroups, GS5_cellSenes_KEGG
normalized, log2CPM, rowClust
Group
CTRL
wtR-LR
mutR-LR
HR
4
2
0
-2
-4

B

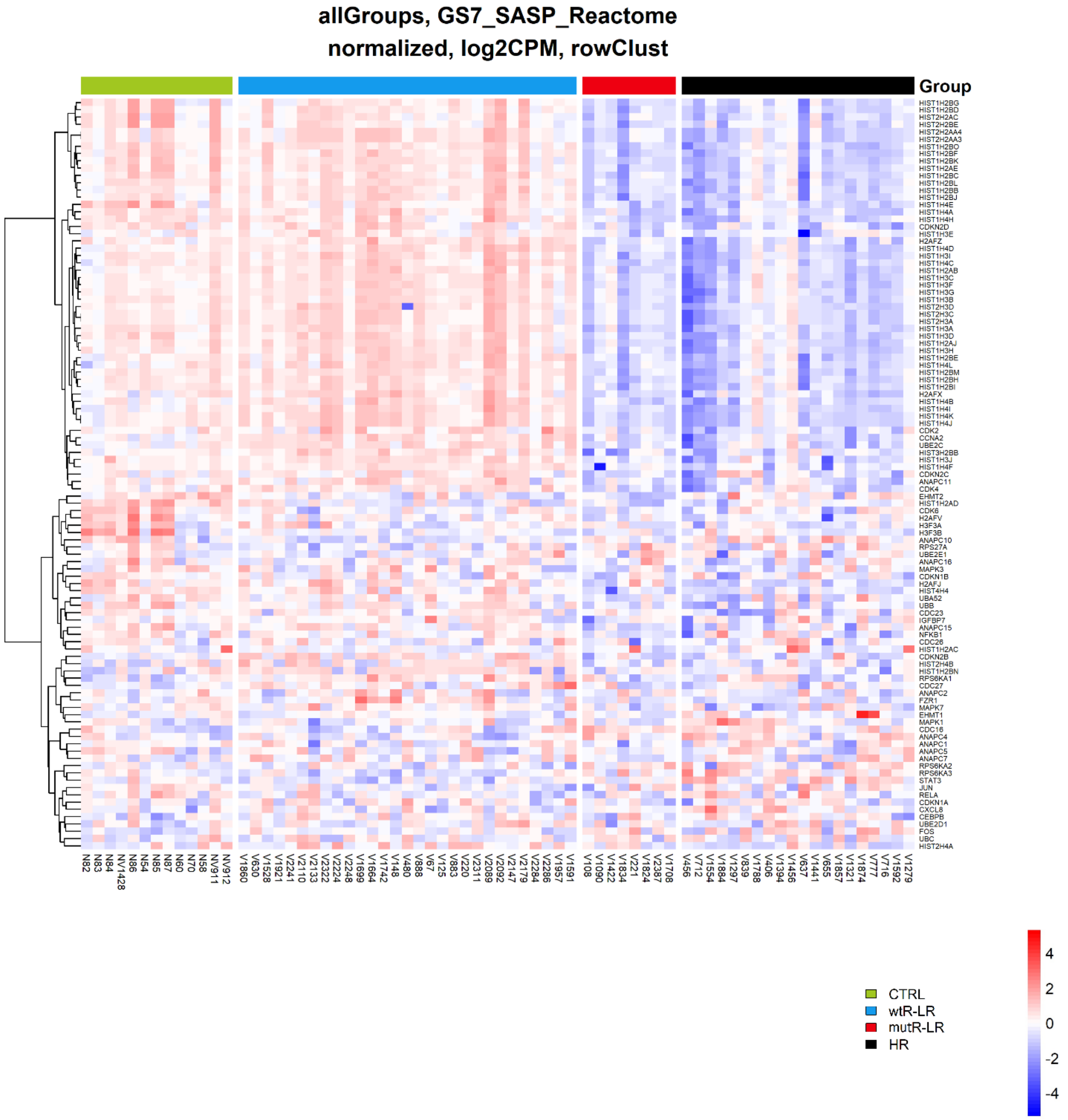
allGroups, GS7_SASP_Reactome
normalized, log2CPM, rowClust
Group
CTRL
wtR-LR
mutR-LR
HR
4
2
0
-2
-4

C

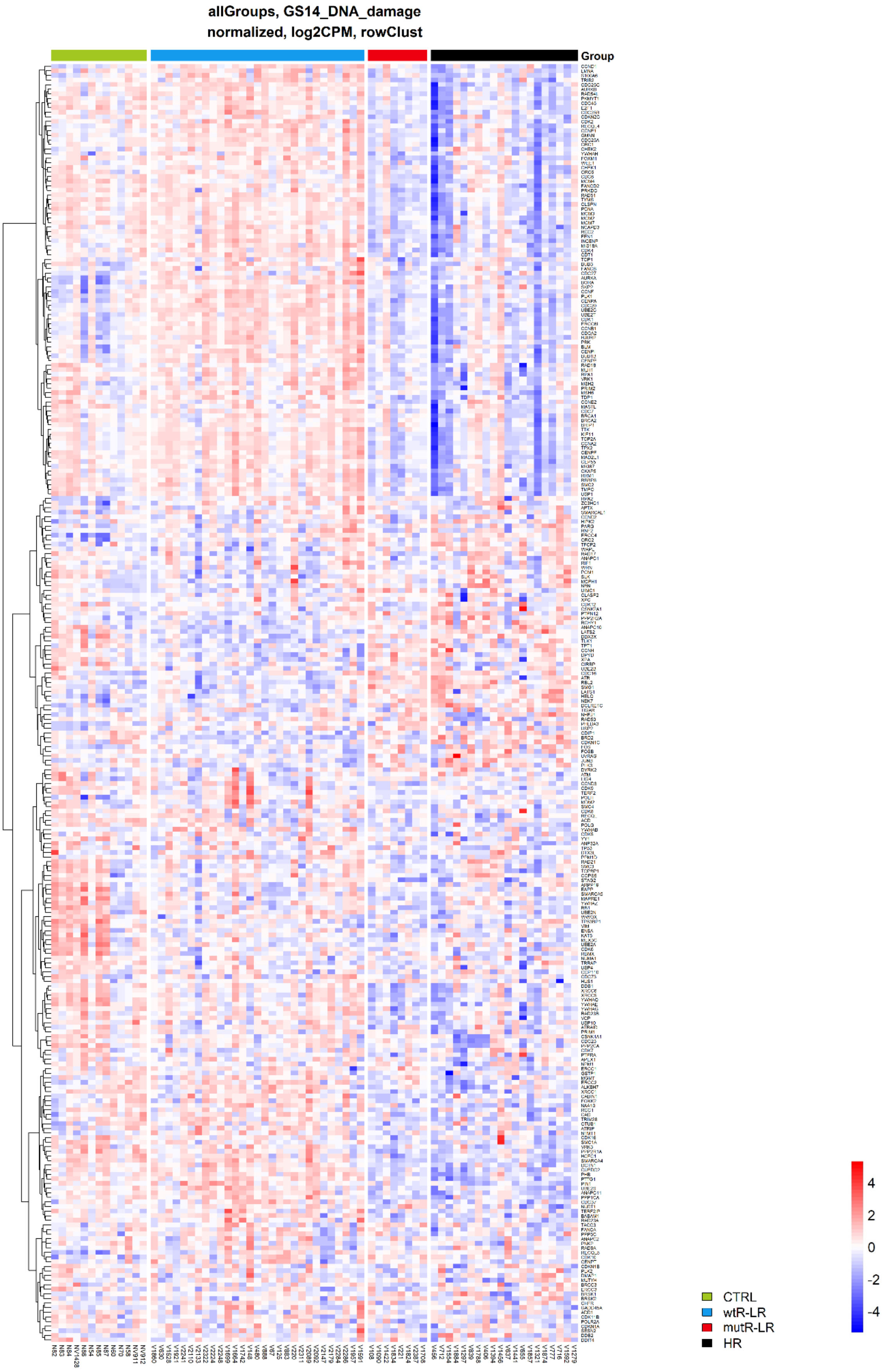
allGroups, GS14_DNA_damage
normalized, log2CPM, rowClust
Group
CTRL
wtR-LR
mutR-LR
HR
4
2
0
-2
-4

SI 20: Dysregulated expression profiles in selected GSEA pathways. Heatmaps show the expression profiles between CD34+ cells from healthy controls (CTRL), wtR-LR, mutR-LR and HR patients in A) Cellular senescence (KEGG), B) SASP (Reactome), C) DNA damage. The red color indicates upregulation, blue color downregulation of gene expression, and the color intensity indicates the level of differential expression.

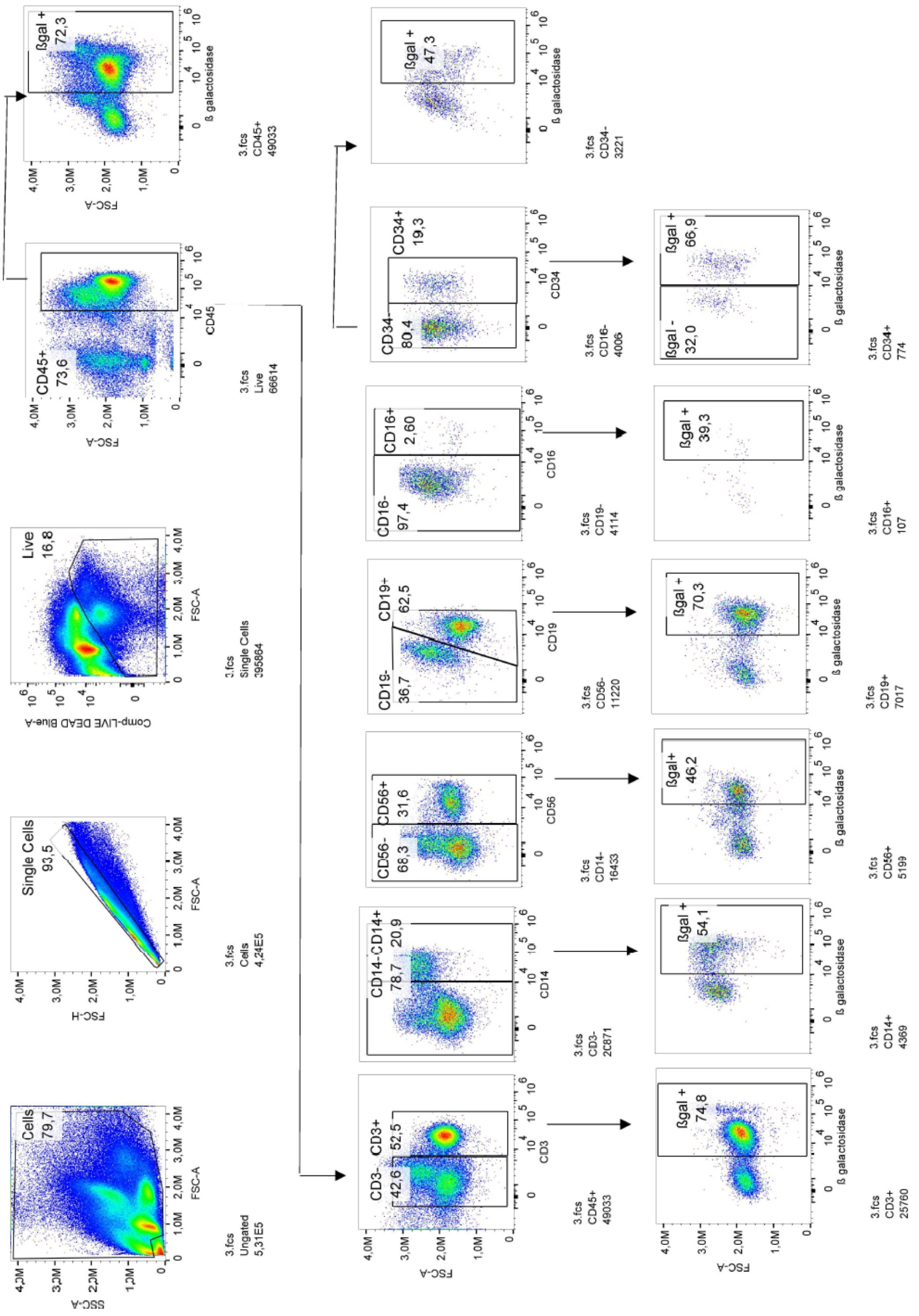

SI 21. Representative example of a gating strategy. The numbers in rectangles indicate the percentage of gated cells.
